# CCL2 supports human hepatocytes long-term expansion for bioartificial liver therapy to relieve acute liver failure and extrahepatic complications

**DOI:** 10.7150/ijbs.115293

**Published:** 2025-10-20

**Authors:** Zibin Zhan, Xuewen Liu, Min Zeng, Zehua Li, Yue Zhang, Xueyan Qiao, Xinming Li, Xianfeng Xia, Kunhao Bai, Fanhong Zeng, Yi Gao, Jun Weng

**Affiliations:** 1 Department of Hepatobiliary Surgery II, Zhujiang Hospital, Southern Medical University, Guangzhou, China.; 2 Guangdong Provincial Research Center for Artificial Organ and Tissue Engineering, Guangzhou Clinical Research and Transformation Center for Artificial Liver, Institute of Regenerative Medicine, Zhujiang Hospital, Southern Medical University, Guangzhou, China.; 3 Department of Endoscopy, Sun Yat-sen University Cancer Center, State Key Laboratory of Oncology in South China, Guangdong Provincial Clinical Research Center for Cancer, Guangzhou, China.; 4 The Second Affiliated Hospital, The State Key Laboratory of Respiratory Disease, Guangdong Provincial Key Laboratory of Allergy & Clinical Immunology, Guangzhou Medical University, Guangzhou, China.; 5 Department of Ultrasound, The Second Affiliated Hospital of Guangzhou Medical University, Guangzhou, China.; 6 Department of Radiology, Zhujiang Hospital, Southern Medical University, Guangzhou, China.; 7 Department of Surgery and State Key Laboratory of Digestive Disease, Institute of Digestive Disease, The Chinese University of Hong Kong, Hong Kong SAR, 999077, China.; 8 Department of Pathology and State Key Laboratory of Liver Research, The University of Hong Kong, Hong Kong.

**Keywords:** acute liver failure, CCL2, hepatocyte proliferation, bioartificial liver, hepatic encephalopathy

## Abstract

The lack of expandable human hepatocytes in vitro hampers the clinical application of the bioartificial liver. Previous studies have shown that chemical cocktails containing growth factors can support long-term expansion of hepatocytes through dedifferentiation. Here, it is revealed that chemokine (C-C motif) ligand 2 (CCL2) is a key factor in liver regeneration. CCL2 could promote the long-term expansion (over 30 passages) of human primary hepatocytes and enhancing their proliferative efficiency. Subsequently, CCL2-mediated proliferation of hepatocytes can effectively expand in vitro, and repopulate the liver of Fah^-/-^ mice following 2-(2-nitro-4-trifluoromethylbenzyol)-1,3- cyclohexanedione (NTBC) withdrawal. Further studies revealed that CCL2-mediated hepatocyte proliferation could yield a sufficient number of highly active and well-functioning hepatocytes, crucial for supporting Bioartificial liver (BAL) therapy in treating acute liver failure (ALF) in a porcine model. Mechanically, BAL therapy effectively suppresses inflammatory responses, promotes liver regeneration, and subsequently protects extrahepatic organs, leading to improved survival rates in ALF porcine models.

## Introduction

Acute liver failure (ALF) is a severe liver injury caused by multiple factors, leading to severe impairment of liver functions, including synthesis, detoxification, metabolism, and biotransformation^1^. It manifests as a clinical syndrome characterized by jaundice, coagulation dysfunction, hepatorenal syndrome and hepatic encephalopathy. Bioartificial liver (BAL) therapy represents the most promising approach for the treatment of liver failure^2^. The core objective of BAL therapy is to metabolize toxic substances associated with liver failure, improve the internal environment and promote liver regeneration^3^. BAL therapeutic outcomes are predominantly influenced by both the viability of transplanted cells and the bioreactor.

A lack of appropriate human hepatocyte sources still hinders the clinical translation of BAL. Inducing stem cells to differentiate into hepatocytes is a promising approach for obtaining seed cells, although the cost and efficiency associated with this process cannot be ignored. Katsuda et al proposed the use of a chemical cocktail to convert rodent primary hepatocytes into proliferative hepatocytes *in vitro*^4^ with hepatocyte function retained. Li et al transfected FOXA3 into a liver progenitor cell line to obtain induced liver progenitor cells with hepatocyte function, which were subsequently utilized for BAL therapy in ALF pigs^5^. Moreover, Guo et al demonstrated that IL-6 supports the long-term expansion of hepatocytes *in vitro*^6^. Recently, more researchers have described the culture of hepatocytes via a combination of chemicals, growth factors, and conditioned medium. These findings prompted us to explore whether a more physiological and effective approach exists to induce the expansion of human primary hepatocytes *in vitro* for BAL therapy to target ALF.

In the ALF pathology model, differentiation of hepatocytes to gain proliferative ability is the main approach for liver regeneration. The levels of several small molecules, including EGF, HGF, TNF-α and IL-6, increase rapidly to induce this process^7^. CCL2, also known as monocyte chemoattractant protein-1 (MCP-1), is a chemokine involved in immune cell migration and inflammation. Strikingly, CCL2 expression is significantly upregulated in the livers of ALF patients and mice with ALF, and plays a significant role in liver regeneration^8^.

Here, we combined single-cell and spatial transcriptome data to analyze the processes of hepatocyte development and liver injury regeneration, identifying CCL2 as a key factor in liver regeneration. We further reported that CCL2-mediated proliferation of hepatocytes could provide a sufficient number of highly active and well-functioning hepatocytes to support BAL therapy for the treatment of ALF in a porcine model. BAL therapy effectively suppresses inflammatory responses, promotes liver regeneration, and thereby protects extrahepatic organs and improves the survival rate in ALF porcine models.

## Material and Methods

The detailed experimental procedures are provided in the supplemental materials and methods.

## Results

### CCL2 promotes human primary hepatocytes expansion *in vitro*

**Figure 1:**
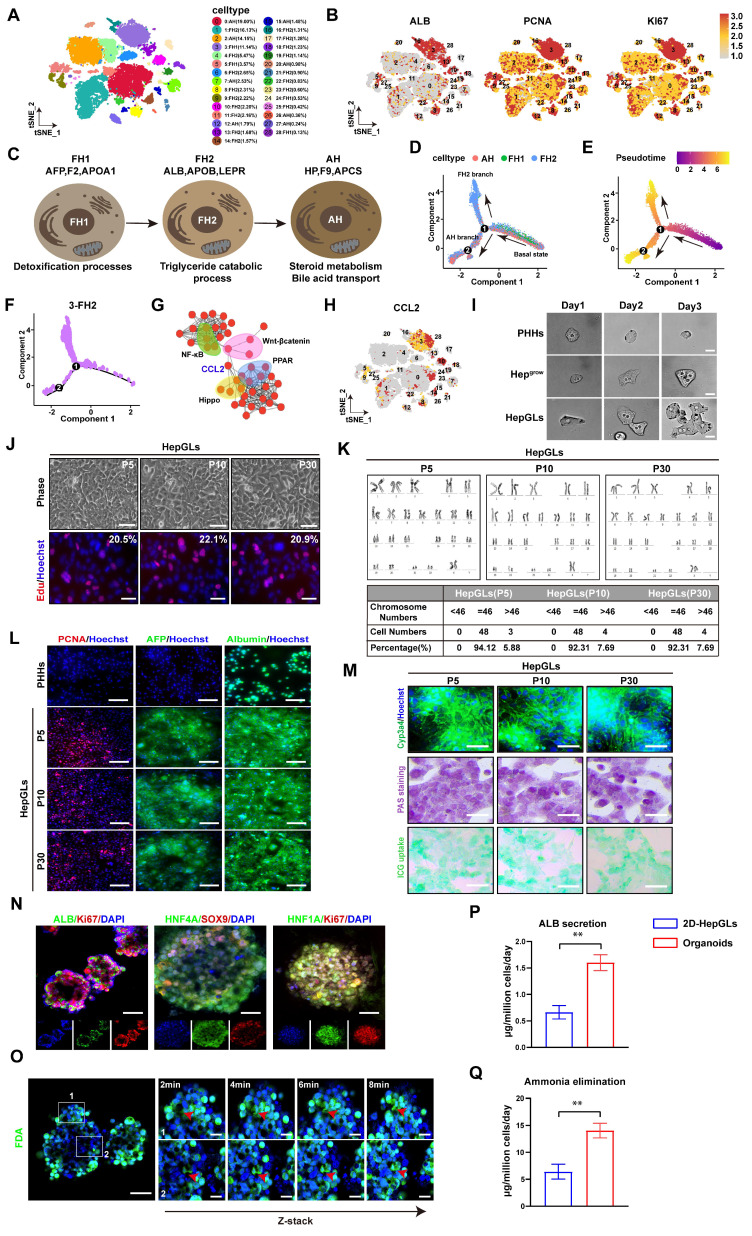
** CCL2 promotes human primary hepatocyte expansion in vitro.** (A) t-SNE visualization of all integrated single-cell transcriptomic data of foetal and adult human hepatic cells. (B) Feature plots of specific marker genes. (C) Characteristic genes induced at each stage of hepatocyte differentiation and corresponding pathway. (D) Pseudotime analysis revealed the bifurcation of hepatocyte trajectory during development. (E) Pseudotime analysis revealed the bifurcation of hepatocyte trajectory during development, with the pseudotime values color-coded. (F) Pseudotime analysis revealed the hepatocyte trajectory during development in Cluster 3. (G) Interaction plot of differentially expressed genes in Cluster 3. (H). Feature plots of CCL2. (I) Time-lapse imaging for 3 days (Scale bars, 100μm). (J) Brightfield photographs and Edu staining images of cells from HepGLs cultured to day 5 in different passages (Scale bars, 100μm). (K) The karyotype analysis of HepGLs at different passages. (L) Immunofluorescence staining for PCNA, AFP, and ALB in PPHs and HepGLs at different passages (Scale bars, 50µm). (M) Immunofluorescence staining for Cyp3a4, bright field photograph of glycogen staining and ICG uptake in HepGLs at different passages. (N) Immunofluorescence images of ALB, Ki67, HNF4A, SOX9 and HNF1A in hepatic organoids (Scale bars, 100µm). (O) Magnified panels (right) correspond to specific outlined regions (left). Red arrows indicate bile canaliculi convergence with biliary cyst (Scale bars, 50µm). (P-Q) ALB secretion(P) and ammonia elimination(Q) of 2D-HepGLs and organoids (n=5 per group). The data represent the mean ± SEM. Statistical significance was assessed by two-tailed Student's t-test and two-way ANOVA. *p < 0.05, **p < 0.01, and ***p < 0.001; n.s., not significant.

Hepatocyte dedifferentiation is the main approach by which hepatocytes obtain proliferative ability in liver regeneration. To screen the key molecules involved in hepatocyte dedifferentiation and differentiation, single-cell transcriptomes from different stages of human liver development were selected and analyzed. Using the t-distributed stochastic neighbor embedding (t-SNE) algorithm, we successfully identified and delineated the principal cell types composing the liver (Fig. 1A, Fig. S1A). We subsequently analyzed the expression patterns of the relevant genes in the single-cell transcriptomes. The expression of genes associated with liver regeneration, such as PCNA, Ki67, AFP and ALB, was enriched in Cluster 3 (Fig. 1B). Next, we depicted the developmental trajectory of each cell type. Principal component analysis (PCA) revealed three stages of hepatocyte development^9^: fetal hepatocyte stages 1 and 2 (FH1, FH2) and adult hepatocyte (AH) stages. Each stage exhibits discernible transcriptional changes indicative of distinct cellular states, implying that hepatocytes undergo progressive functional maturation corresponding to the development of hepatic activity during organogenesis (Fig. 1C). Furthermore, we mapped hepatocytes along pseudotemporal trajectories and observed a branching point in the hepatocyte trajectory at a specific node (labeled “1” in Fig. 1D). The branch to the right of the node contains cells mainly from stage FH1. After node 1, this branch is further divided into two branches. The upper branch is composed mainly of cells from the FH2 stage, whereas the lower branch consists primarily of AH hepatocytes (Fig. 1D-E, Fig. S1B). Furthermore, we investigated the pseudotemporal trajectories of each cell type and found that Cluster 3 accumulated in the FH2 stage after node 1. Therefore, we selected Cluster 3 for further investigation (Fig. 1F).

We subsequently examined the DEGs that exhibited increased expression in Cluster 3 and identified a significant association between CCL2 expression and regenerative pathways such as the Hippo/Yap and Wnt/β-catenin pathways(Fig. 1G-H, Fig. S1C).Previous studies have reported that the ability of hepatocytes to regain their proliferative ability via dedifferentiation plays a crucial role in liver injury-induced liver regeneration^10^. TNFα and TGF-β have been identified as important inflammatory cytokines that enhance liver regeneration^4^. Since CCL2 is an inflammatory chemokine, we aimed to investigate its role in liver regeneration. We analyzed gene expression in patients and mice with ALF and detected abnormally high expression of CCL2 (Fig. S1D-K). We subsequently explored the spatial transcriptome data and investigated the co-expression of CCL2 with that of genes related to liver regeneration and dedifferentiation in human and mouse APAP-induced ALF (Fig. S1L-M). Furthermore, we constructed APAP induced mice models (Fig. S2A-C) and further confirmed high-CCL2 hepatocytes displayed stronger proliferative activity (Fig. S2D). In addition, we conducted immunofluorescence staining on liver tissues from humans with APAP-induced ALF recovery, and the results revealed strong co-expression of CCL2 with Ki67, YAP, and HNF4A in hepatocytes (Fig. S2E). These confirmed CCL2 could mediate hepatocytes proliferation in ALF. Previous studies demonstrated that hepatocytes could secrete CCL2 via CCR2 to activate immune cells to mediate liver regeneration^11^ and we further confirmed that CCL2 secretion by hepatocytes could recruits myeloid cells that may synergistically enhance liver repair during ALF (Fig. S3A-D). Furthermore, we employed a chemical-based strategy to induce hepatocyte dedifferentiation and stimulate hepatocyte growth* in vitro*^4^ and named the obtained cells Hep^grow^. Furthermore, we introduced CCL2 to examine its potential to promote proliferation, and the cells were named Hep^growccl2^ (HepGLs) (Fig. S4A). We then performed time-lapse imaging to track the fates of sparsely inoculated HepGLs. Notably, we found that CCL2 supports the reprogramming of hepatocytes into a more proliferative state (Fig. 1I, Fig. S4B). On day 14, the proliferating cells presented a distinct epithelial morphology characterized by a high nucleus/cytoplasm ratio (Fig. S4C). Notably, HepGLs induced by CCL2 presented the highest proliferative capacity compared with those in the other groups (Fig. S4D). The HepGLs could be passaged more than 30 times without any apparent morphological changes (Fig. 1J). Moreover, the cumulative cell number consistently increased from 1x10^4^ to approximately 4-5x10^5^ within a 5-day period, with no significant differences observed among the different passages (Fig. S4E-F). The cells did not exhibit any nuclear aberrations or chromosomal abnormalities after multiple passages *in vitro* (Fig. 1K). Immunofluorescence staining revealed that HepGLs at passages P5, P10 and P30 presented stable and high expression of albumin and AFP, as well as of the cell cycle marker PCNA (Fig. 1L). RT-PCR analysis confirmed the sustained high expression levels of hepatocyte functional genes in HepGLs at P5, P10 and P30(Fig. S4G). The ability of HepGLs at all passages to eliminate ammonia and secrete albumin was slightly inferior to that of primary human hepatocytes (PHHs) (Fig. S4H-I). PAS staining, indocyanine green (ICG) uptake assays, and immunofluorescence staining of Cyp3a4 expression revealed that HepGLs at P5, P10, and P30 presented mature hepatocyte functions similar to those of PHHs (Fig. 1M). Moreover, we discovered that HepGLs may achieve this function through the activation of the Hippo/YAP signaling pathway by CCL2 (Fig. S5A-F). To further explore this mechanism and the hepatic functions of HepGLs, we employed a human liver organoid model. The organoids displayed normal hepatocyte morphology (Fig. S5G-I) and function after 14 days induction. Immunofluorescence analysis confirmed high expression levels of mature hepatocyte markers and proliferation markers in the organoids (Fig. 1N). Normal bile secretion was observed in the organoids (Fig. 1O). Notably, the ability of the organoids to secrete albumin and eliminate ammonia was significantly greater than that of the 2D-HepGLs (Fig. 1P-Q). We further interrogated HepGLs mediated liver regeneration mechanism using the YAP inhibitor Dobutamine^12^, ^13^ and CCR2 blockade (Fig. S4J-L) to confirm CCL2 promotes hepatocyte proliferation and functional enhancement through activation of the Hippo/YAP signaling pathway, supporting its critical role in liver regeneration (Fig. S5F, S5J-M).

Furthermore, we utilized a CCl_4_-induced liver fibrosis mouse model to evaluate the therapeutic potential of HepGLs in liver failure (Fig. S6A) and found that mice treated with HepGLs showed partial amelioration of CCl_4_-induced liver fibrosis and inflammation (Fig. S6B-E).

### HepGLs repopulate the livers of Fah^⁻/⁻^ mice

**Figure 2:**
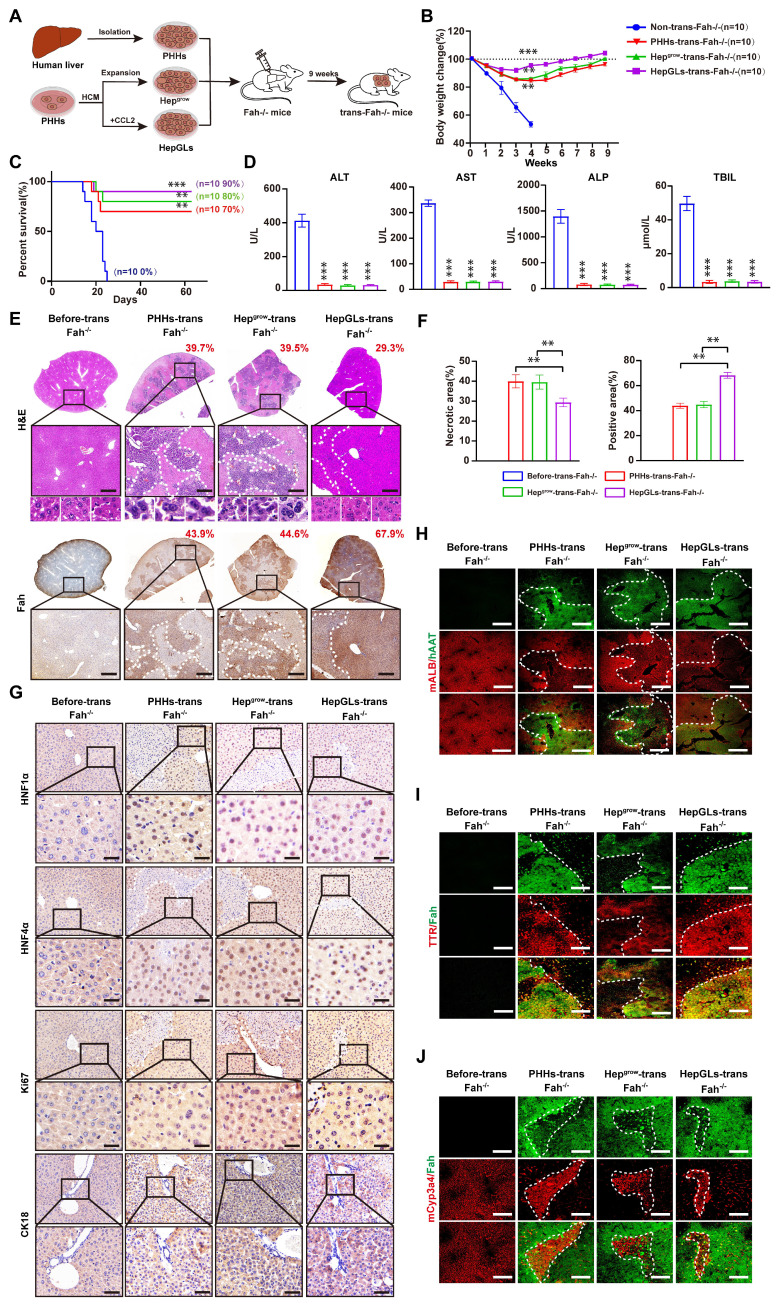
** HepGLs repopulate the livers of Fah**^-/-^** mice.** (A) Schematic diagram of the experimental procedure: hepatocytes were transplanted into the liver of Fah^⁻/⁻^ mice, and the repopulated livers were obtained for further experiments 9 weeks later. (B) Body weight change of Fah^⁻/⁻^ mice receiving vehicle, PPHs, Hep^grow^ and HepGLs (n=10 mice each group). (C) Survival curves of the animals in the four groups. (D) Serum levels of ALT, AST, ALP and TBIL in the Fah^⁻/⁻^ mice after transplantation. (E) Liver sections of Fah^⁻/⁻^ mice before or 9 weeks after transplantation were stained with H&E and analyzed by IHC for Fah levels (Scale bars=100μm; n=5 per group). (F) Quantify the necrotic area and the repopulated area for each group. (G) IHC staining of HNF1α, HNF4α, Ki67 and CK18 in liver sections of Fah^⁻/⁻^ mice before or 9 weeks after transplantation (Scale bars=100μm; n=5 per group). (H-J) Immunofluorescence staining of ALB, AAT, TTR, Cyp3a4 and Fah in liver sections of Fah^⁻/⁻^ mice before or 9 weeks after transplantation (Scale bars=200μm; n=5 per group). The data represent the mean ± SEM. Statistical significance was assessed by two-tailed Student's t-test and two-way ANOVA. *p < 0.05, **p < 0.01, and ***p < 0.001; n.s., not significant.

To examine the ability of HepGLs to treat ALF *in vivo*, we performed a Fah^⁻/⁻^ mouse rescue experiment. Fah^⁻/⁻^ mice require the administration of 2-(2-nitro-4-trifluoromethylbenzyol)-1,3- cyclohexanedione (NTBC) for survival^14^. Upon NTBC withdrawal, Fah^⁻/⁻^ mice experience ALF and die within approximately one month, making them an ideal model for assessing hepatocyte proliferation and function *in vivo*^15^. To evaluate the hepatocytic functions of HepGLs *in vivo*, we performed intrasplenic injections of PHHs, Hep^grow^, or HepGLs to repopulate the livers of Fah^⁻/⁻^ mice (Fig. 2A). After NTBC withdrawal, all 10 non-transplanted mice experienced continuous weight loss and eventually died within 4 weeks (Fig. 2B-C). In contrast, mice that received PHHs, Hep^grow^, or HepGLs exhibited weight gain after four weeks and had a low mortality rate. During the nine-week observation period, the mortality rates of the mice that received PHHs, Hep^grow^, and HepGLs were 70%, 80%, and 90% respectively (Fig. 2B-C). The levels of AST, ALT, ALP, and total bilirubin in the serum of non-transplanted mice significantly increased, whereas these levels nearly returned to normal in the mice after transplantation (Fig. 2D).

Nine weeks post-transplantation, the livers that received PHHs, Hep^grow^, and HepGLs repopulated a significant portion of the liver, with the HepGLs group exhibiting the highest reconstruction efficiency. Histological examination confirmed minimal liver damage and clear nuclear division in hepatocytes after transplantation (Fig. 2E-F). Immunohistochemistry revealed prominent positive staining for HNF1α, HNF4α, Ki67, and CK18 in repopulated PHHs, Hep^grow^, and HepGLs in Fah^⁻/⁻^ mice, with the HepGLs group demonstrating the highest percentage of positive staining (Fig. 2G). Similarly, immunofluorescence analysis showed significant positive staining for albumin, TTR, and Cyp3a4 in repopulated PHHs, Hep^grow^, and HepGLs in Fah^⁻/⁻^ mice, with the HepGLs group exhibiting the greatest proportion of positive staining (Fig. 2H-J). These findings demonstrate that HepGLs have the capacity to repopulate the livers of Fah-/- mice and effectively alleviate ALF more efficiently than PHHs do. HepGLs can effectively expand *in vitro*, exhibit hepatocytic functions, and repopulate the livers of Fah^⁻/⁻^ mice, thereby preventing ALF.

### Large-scale culture of HepGLs and PPHs in the bioreactor for BAL therapy

**Figure 3:**
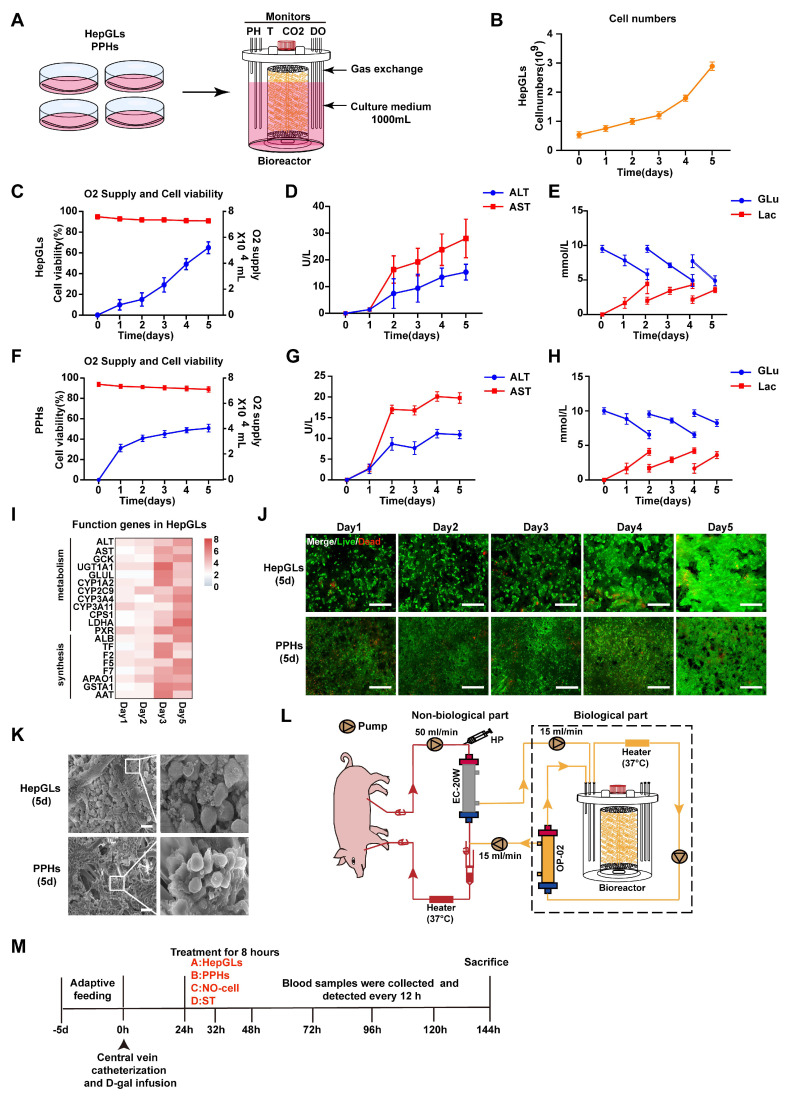
** Large-scale culture of HepGLs and PPHs in a bioreactor for BAL therapy.** (A) Schematic diagram of the large-scale expansion procedure for PPHs and HepGLs. (B)Cell numbers of HepGLs culture from 0 to 5 d (n=3 per time point). (C) Oxygen supply (Red) and cell viability (Blue) of HepGLs culture from 0 to 5 d (n=3 per time point). (D) ALT and AST levels in HepGLs cultured from 0 to 5 d (n=3 per time point). (E) Glucose and lactate levels in HepGLs cultured from 0 to 5 d (n=3 per time point). (F) Oxygen supply (Red) and cell viability (Blue) of PPHs cultured from 0 to 5 d (n=3 per time point). (G) ALT and AST levels in PPHs cultured from 0 to 5 d (n=3 per time point). (H) Glucose and lactate levels in PPHs cultured from 0 to 5 d (n=3 per time point). (I) HepGLs' functional gene expression after 5 days of culture (n=3 per time point). (J) Live/dead detection of the viability of HepGLs and PPHs from 1 to 5d, which remained more than 80% viable (Scale bars = 200 µm, n=3 per group). (K) Scanning electron microscopy (SEM) revealed that the HepGLs (5 days) and PPHs (5 days) attached to the fibers well (Scale bars=100µm). (L) Schematic diagram of the FSB BAL therapy system. HP, heparin pump; EC-20W, OP-02, plasma separator. (M) Experimental timeline showing the BAL treatment in ALF pigs. The data represent the mean ± SEM. Statistical significance was assessed by two-tailed Student's t-test and two-way ANOVA. *p < 0.05, **p < 0.01, and ***p < 0.001; n.s., not significant.

Obtaining a large number of cells (10^9^ to 10^10^) for BAL therapy is a significant challenge. The fiber scaffold bioreactor (FSB) we designed effectively sustains the viability and functionality of hepatocytes at a high density (Fig. 3A). We employed an FSB to culture and expand HepGLs and primary porcine hepatocytes (PPHs). Over a span of 5 days, the HepGLs exhibited a remarkable expansion from an initial count of 4-5 × 10^8^ to 3-4 × 10^9^ within the confines of the FSB (Fig. 3B). This bioreactor ensures an ample supply of oxygen and nutrients, resulting in notable cell viability exceeding 80%, accompanied by minimal cellular damage (Fig. 3C-D). The variations in glucose and lactate levels within the culture medium serve as indicators of the cellular proliferation rate (Fig. 3E). Furthermore, a population of 3-4×10^9^ PPHs was cultivated within the bioreactor as a control, which provided favorable nutritional conditions and minimized cellular damage, as evidenced by ALT and AST levels, with a commendable cell survival rate surpassing 80% (Fig. 3F-G). The fluctuations in glucose and lactate levels are graphically represented in Fig.3H. Throughout the 5-day culture period, both HepGLs and PPHs consistently presented high expression levels of hepatocytic functional genes, such as GCK, CYP1A2 and F5(Fig. 3I, Fig. S7A-E). The bioreactor promoted strong adhesion and growth of both HepGLs and PPHs while maintaining high cell viability and proliferation rates throughout the 5-day culture period (Fig. 3J-K). To further assess metabolic functions of HepGLs-BAL**,** we established an in vitro liver failure serum model^16^ simulating acute liver failure and evaluated the metabolic detoxification capacity of the HepGLs-equipped BAL system. After 8h circulation, the HepGLs-BAL demonstrated decreased ammonia levels with increased urea synthesis, indicating robust ureagenesis. Additionally, albumin levels were significantly elevated (Fig. S7F-H), and substantial reductions in total bilirubin (TBIL) and bile acids (TBA) (Fig. S7I-J). No creatinine (Cr) reduction was observed in any group (Fig. S7K). Critically, HepGLs maintained over 80% viability throughout treatment (Fig. S7L). These results confirm that the HepGLs-BAL sustains high viability while providing significant detoxification and synthetic function under ALF.

### BAL treatment of the preclinical porcine model of ALF

**Figure 4:**
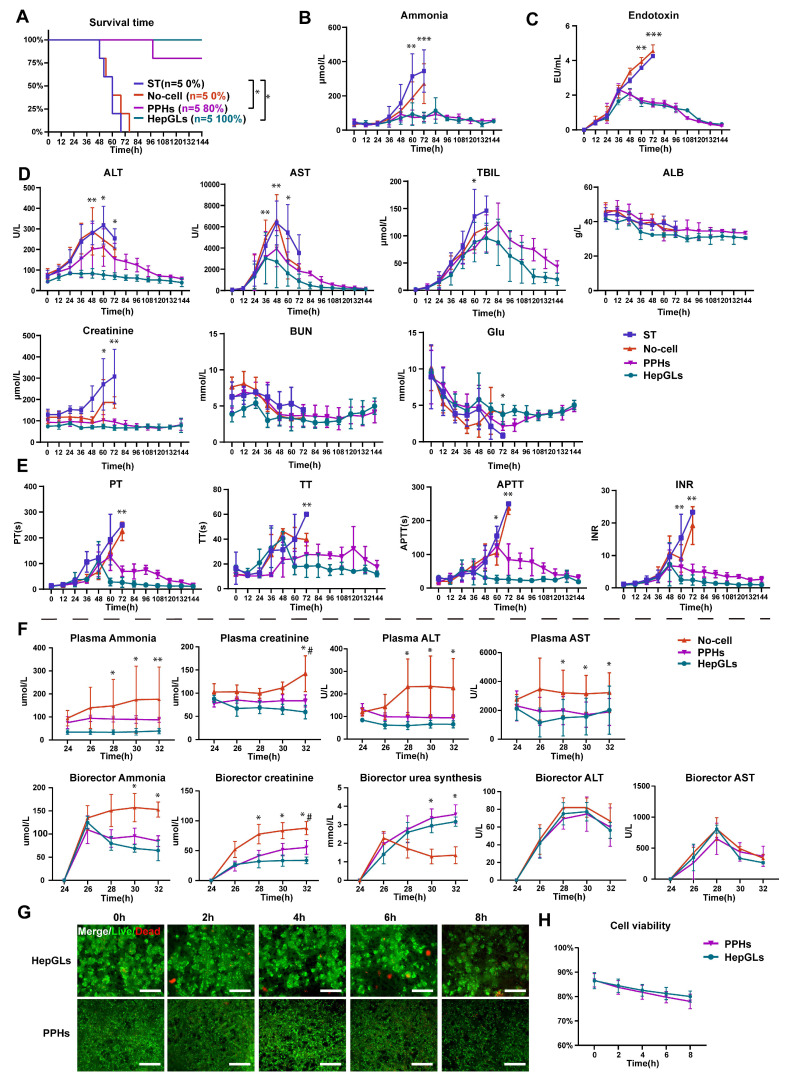
** BAL treatment of the preclinical porcine model of ALF.** (A) Kaplan-Meier survival curve in ST, No-cell, PPHs and HepGLs groups (n=5 per group). (B-C) Plasma ammonia and endotoxin in the four groups during the experiment (n=5 per group). (D) Plasma biochemical indexes in the four groups during the experiment (ALT, AST, TBIL, ALB, Cr, BUN, Glu; n= 5 per group). (E) Coagulation function parameters in the four groups during the experiment (PT, TT, APTT, and INR; n=5 per group). (F) Blood biochemistry indexes in the three groups during treatment in the bioreactor and blood (n=5 per group). (G-H) Live/dead staining and cell viability of PPHs and HepGLs during treatment (n=5 per group). The data represent the mean ± SEM. Statistical significance was assessed by two-tailed Student's t-test and two-way ANOVA. *p < 0.05, **p < 0.01, and ***p < 0.001; n.s., not significant.

Next, we explored the safety and effectiveness of BAL therapy based on HepGLs in animals with ALF. To induce an ALF model in Tibetan miniature pigs, 0.45 g/kg D-galactosamine (D-gal) was administered via the central vein, following established research protocols^16^. The experimental design is depicted in Fig.3L-M and Fig. S5; the animals were subjected to 8 h of treatment (Fig. S7M). Animals that were subjected to BAL therapy presented significantly increased survival rates. Specifically, the survival rates of the HepGLs group (5/5, 100%) and the PPHs group (4/5, 80%) were notably greater than those of the ST group (0/5, 0%) and the No-cell group (0/5, 0%) at the 144-h study end point (Fig. 4A and Table 1). Biochemical analysis revealed that the levels of blood ammonia and endotoxin in the HepGLs group and the PPHs group began to decrease at 48 h, in contrast to those in the ST group and the No-cell group, in which these levels continued to increase until the animals died (Fig. 4B-C). All the animals in the ST group and the No-cell group ultimately developed hepatic encephalopathy (HE), whereas only one animal in the PPHs group and none in the HepGLs group presented evident symptoms of HE. The levels of ALT and AST began to decrease after 48 h, and the most notable decrease was observed in the HepGLs group. The TBIL levels initially increased but eventually normalized in both the HepGLs and PPHs groups after BAL treatment. Additionally, the creatinine level in the ST group significantly increased prior to mortality, suggesting the occurrence of hepatorenal syndrome in these animals. The blood urea nitrogen (BUN) level did not differ significantly among the groups. However, the glucose and ALB levels consistently decreased in all of the groups and subsequently gradually recovered in the two BAL groups, as depicted in Fig.4D. Coagulation function deteriorated in the ST group and the No-cell group, but gradually returned to normal in the two BAL groups (Fig. 4E). These findings suggest the occurrence of coagulation dysfunction in animals with D-gal induced ALF.

Additionally, we assessed various biochemical indicators in both the bioreactor and the blood to evaluate the efficacy of BAL treatment. During the 8-h treatment, the ammonia level in the plasma only slightly increased, whereas the ammonia level in the bioreactor gradually decreased in the two BAL groups compared with that in the No-cell group. These findings suggest that BAL therapy partially substitutes for liver functions and detoxification processes. The creatinine level in both the plasma and bioreactor continuously increased in the No-cell group, whereas that in the two BAL groups was maintained at a lower level, indicating a protective effect on renal function. Furthermore, the two BAL groups presented an increase in urea synthesis, indicating efficient conversion of ammonia to urea. While there were no considerable differences in ALT and AST levels within the bioreactor among the three groups, the plasma levels of ALT and AST were lower in both the HepGLs and PPHs groups than in the No-cell group (Fig. 4F). During the 8 h treatment period, the viability of HepGLs and PPHs remained consistently high, suggesting the sustainability and reproducibility of the BAL system (Fig. 4G-H). In summary, HepGLs-BAL therapy may improve D-gal-induced liver injury and increase the survival rates of pigs.

### BAL therapy relieves liver damage and inflammation and promotes liver regeneration

**Figure 5:**
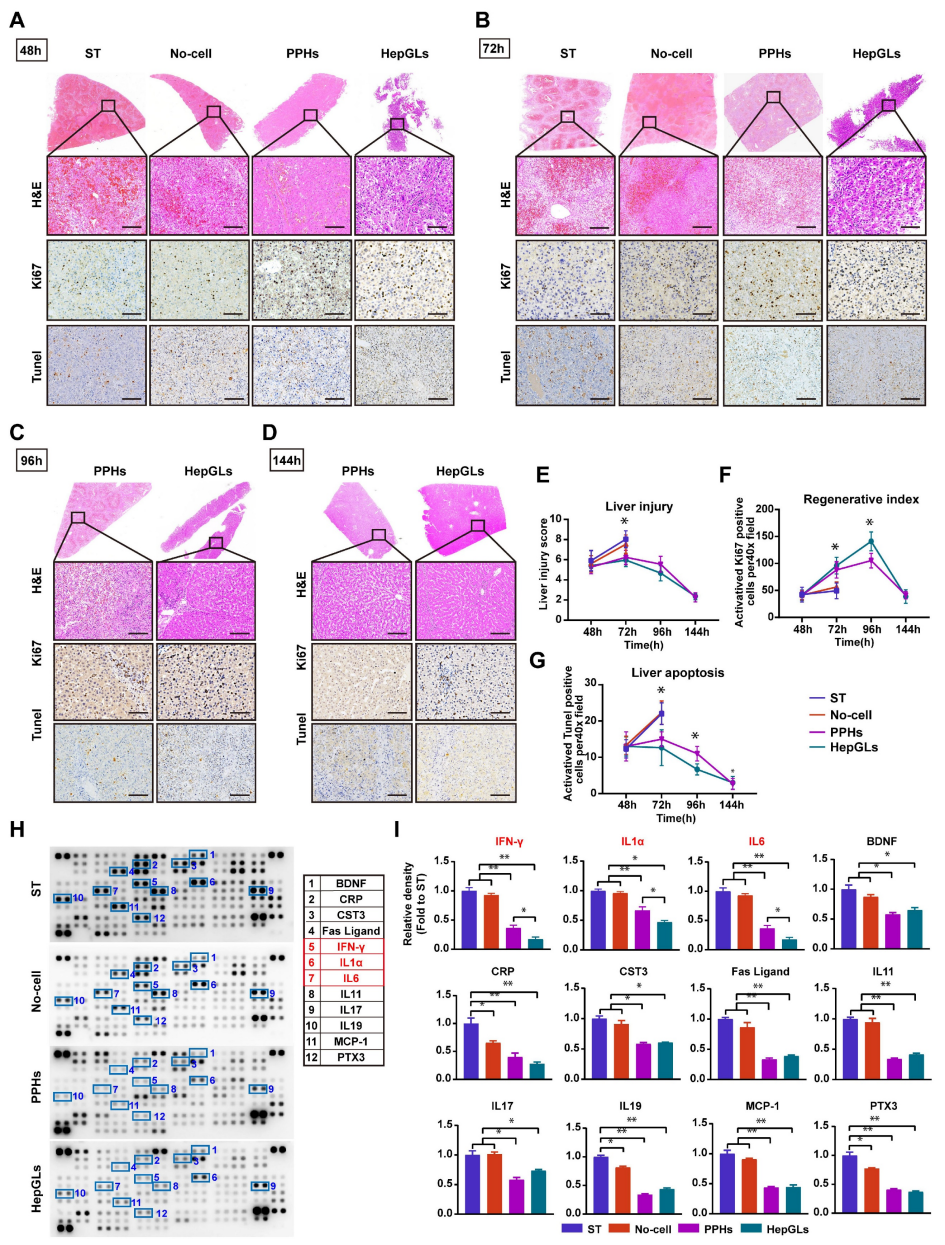
** BAL therapy relieves liver damage and inflammation and promotes liver regeneration.** (A) H&E staining and IHC for Ki67 and TUNEL of liver tissues from the ST, No-cell, PPHs and HepGLs groups at 48 hours after D-gal infusion (Scale bars=100μm; n=5 per group). (B) H&E staining and IHC for Ki67 and TUNEL of liver tissues from the ST, No-cell, PPHs and HepGLs groups at 72 hours after D-gal infusion (Scale bars=100μm; n=5 per group). (C) H&E staining and IHC for Ki67 and TUNEL of liver tissues from the PPHs and HepGLs groups at 96 hours after D-gal infusion (Scale bars=100μm; n=5 per group). (D)H&E staining and IHC for Ki67 and TUNEL of liver tissues from the PPHs and HepGLs groups at 144 hours after D-gal infusion (Scale bars=100μm; n=5 per group). (E) Liver injury score in the four groups at different time points. (F) Regenerative index (Ki-67) in the four groups is quantified by Ki-67 positive cells per 40× high-power field. (G) The liver apoptosis score was quantified by TUNEL-positive cells in the four groups. (H) Plasma samples at 72 hours after D-gal infusion in the four groups were analyzed using the cytokine array kit (Cat. ARY022B) for the detection of 105 different cytokines and chemokines (n=4 per group). (I) Quantification of (H). The data represent the mean ± SEM. Statistical significance was assessed by two-tailed Student's t-test and two-way ANOVA. *p < 0.05, **p < 0.01, and ***p < 0.001; n.s., not significant.

We employed immunohistochemistry and hematoxylin and eosin (H&E) staining to observe the progression of ALF and further identified the effects of BAL therapy in animals with ALF. H&E staining at 48 h revealed hemorrhage, vacuolar changes, and necrosis in the livers across all groups (Fig. 5A). At 72 h, animals in the ST and No-cell groups exhibited extensive necrosis and pronounced inflammation, whereas liver damage was significantly mitigated in the two BAL groups (Fig. 5B). Gradual recovery of liver damage was subsequently observed in the two BAL groups, as illustrated in Fig. 5C and 5D. After 48 h of D-gal infusion, all of the groups presented a notable presence of apoptotic cells, as evidenced by an abundance of positive TUNEL staining. Notably, the two BAL groups displayed reduced apoptosis at 72 h, which continued to decrease over time. Staining for the regenerative marker Ki67 revealed significantly positive cells across all groups at 48 h. The two BAL groups presented a continuous increase in the number of Ki67-positive cells, reaching a peak at 96 h, whereas the ST group and No-cell group presented a comparatively lower regeneration index (P<0.05) (Fig. 5E-F). Surprisingly, compared with the PPHs group, the HepGLs group presented a greater number of Ki67-positive cells at 96h (Fig. 5C, 5G). Immunofluorescence staining revealed strong co-expression of Ki-67 with CK18, ALB and YAP in hepatocytes from the HepGLs group, indicating their dedifferentiation into an immature state (Fig. S7N). This dedifferentiation may contribute to the proliferative capacity for residual liver regeneration.

Acute drug-induced liver failure triggers the release of inflammatory factors following hepatocyte necrosis, leading to an inflammatory storm and subsequent multiorgan dysfunction. Plasma samples collected at 72 h were subjected to dot blot analysis to assess the levels of proinflammatory cytokines. Compared with those in the ST group and the no-cell group, the BAL therapy group presented significantly lower levels of proinflammatory cytokines and chemokines, such as IFN-γ, IL-1a, IL-6, CRP, FasL, IL-11, IL-17, IL-19, MCP-1, and PTX3. Furthermore, the HepGLs group presented markedly lower levels of IFN-γ, IL-1a, and IL-6 than the PPH group did (Fig. 5H-I). As depicted in Figure 5I, Cystatin C (CST3), a marker that is more sensitive and specific than creatinine for estimating the glomerular filtration rate^17^, were significantly elevated in the animals in the ST and No-cell groups, indicating renal failure following D-gal infusion. The expression of brain-derived neurotrophic factor (BDNF), which belongs to the neurotrophic factor protein family and can cross the blood-brain barrier^18^, was significantly elevated in the ST and no-cell groups. This increase in BDNF expression may be associated with increased reactivity following hepatic encephalopathy. Taken together, these findings suggest that BAL therapy not only promotes liver regeneration but also alleviates the inflammatory response, indicating its potential effectiveness in treating ALF.

### BAL therapy prevents intestinal barrier disruption and renal failure in porcine ALF

**Figure 6:**
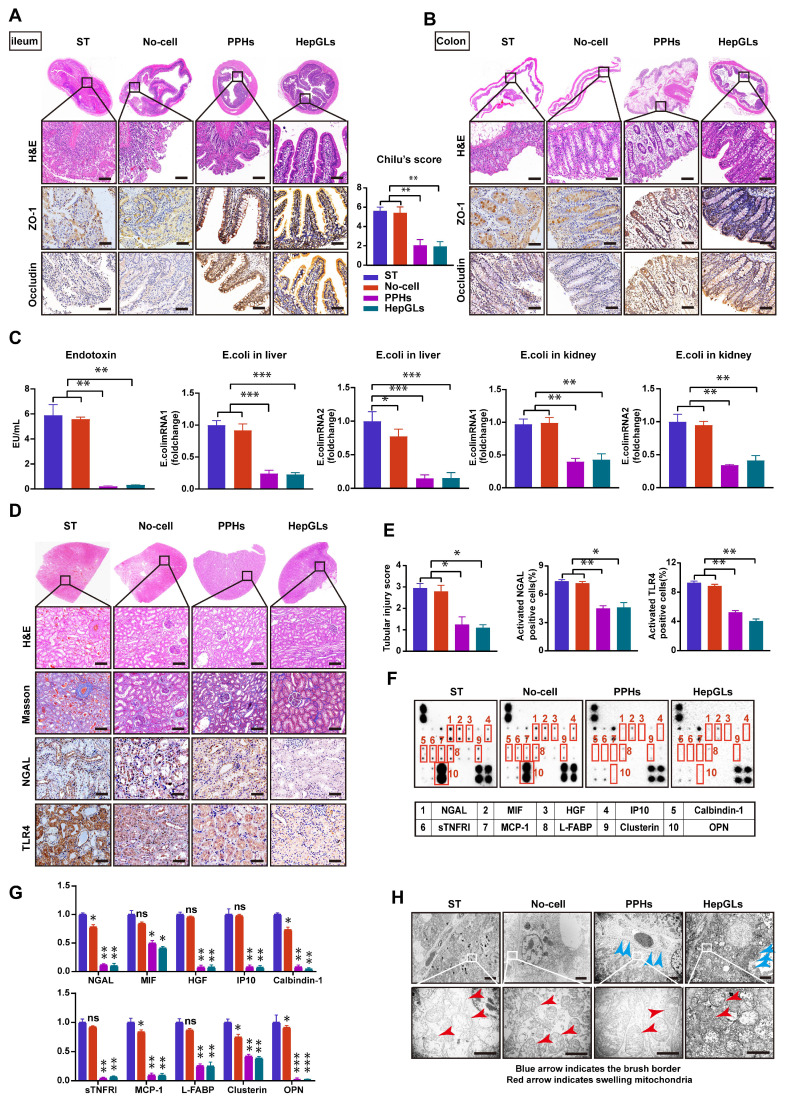
** BAL therapy prevents intestinal barrier disruption and renal failure in porcine ALF.** (A) H&E staining and IHC for ZO-1 and Occludin of ileum tissues from the ST, No-cell, PPHs and HepGLs groups at the endpoint(left). Chilu's score for ileum injury(right) (Scale bars=100μm; n=5 per group). (B) H&E staining and IHC for ZO-1 and Occludin of colon tissues from the ST, No-cell, PPHs and HepGLs groups at the endpoint (Scale bars=100μm; n=5 per group). (C) Plasma endotoxin and E. coli mRNA expression in the liver and kidney (n=5 per group). (D) H&E staining, Masson staining and IHC for NGAL and TLR4 of kidney tissues from the ST, No-cell, PPHs and HepGLs groups at the endpoint (Scale bars=100μm; n=5 per group). (E) Tubular injury score, NGAL and TLR4 positive cells in the four groups (n=5 per group). (F) AKI-associated plasma protein assay at 72 h (n=4 per group). (G) Quantification of (F). (H)TEM observation of the kidney at the endpoint (red arrow indicates swelling mitochondria; blue arrow indicates the brush border; Magnification Bar = 500 nm). The data represent the mean ± SEM. Statistical significance was assessed by two-tailed Student's t-test and two-way ANOVA. *p < 0.05, **p < 0.01, and ***p < 0.001; n.s., not significant.

ALF patients often experience intestinal barrier disruption, which can cause bacterial translocation and exacerbate liver injury and inflammation. Therefore, we next examined the intestine via H&E staining and detected profound cellular damage, a heightened presence of inflammatory cells, and extensive separation of glands in the epithelium of the ileum and colon in the ST and No-cell groups; these effects were not detected in the two BAL groups. As shown in Fig. 6A, the animals in the ST and No-cell groups presented higher injury scores. The expression of the key transmembrane tight junction proteins ZO-1 and occluding, was significantly increased in the two BAL groups (Fig. 6A-B). Gut leakage occurs when the gap between intestinal mucosal cells widens. Simultaneously, endotoxins secreted by *Escherichia coli*(*E. coli*) enter the bloodstream through enlarged gaps in the intestinal mucosa, leading to a systemic inflammatory response^19^. Compared with those in the two BAL groups, the plasma endotoxin levels and *E. coli* mRNA expression levels in the liver and kidneys were significantly greater in the ST and No-cell groups, indicating that BAL therapy prevents intestinal barrier disruption (Fig. 6C).

Acute kidney failure, a prominent complication of ALF, is associated with a high mortality rate. We assessed the plasma creatinine levels and observed a significant reduction in those of the two BAL groups at 72 h following D-gal infusion compared with those of the ST and No-cell groups (Fig. 4D). H&E staining revealed necrosis and shedding of renal tubular epithelial cells, along with tubular dilation, protein casts, and the presence of inflammatory cells in the ST and No-cell groups. However, the glomerular morphology appeared generally normal across all of the animal groups. Masson staining analysis revealed no significant fibrosis in any of the groups. As a prominent biomarker of kidney injury, neutrophil gelatinase-associated lipocalin (NGAL) levels were significantly lower in the two BAL groups. Toll-like receptors (TLRs) play crucial roles in the inflammatory damage process of acute kidney injury (AKI). Studies have demonstrated that the inhibition of Toll-like receptor 4 (TLR4) expression has a notable protective effect on renal dysfunction^20^, ^21^. Notably, TLR4 expression levels were significantly lower in the two BAL groups than in the ST and no-cell groups (Fig. 6D-E).

To further explore potential factors associated with AKI, we applied an AKI-related antibody array, focusing on the levels of 20 cytokines. The results revealed a significant decrease in NGAL, macrophage migration inhibitory factor (MIF), hepatocyte growth factor (HGF), interferon gamma-induced protein 10 (IP10), calcium-binding protein 1 (calbindin-1), soluble tumor necrosis factor receptor I (sTNFRI), monocyte chemoattractant protein-1 (MCP-1), liver fatty acid-binding protein (L-FABP), clusterin, and osteopontin (OPN) levels in the two BAL groups (Fig. 6F-G). Transmission electron microscopy (TEM) revealed significant dilation of renal tubules, a reduced brush border, mitochondrial swelling, and cristae dissolution in the ST and No-cell groups. In contrast, the two BAL groups presented mild renal tubular injury, with preserved brush borders, mild mitochondrial swelling, and intact mitochondrial cristae (Fig. 6H). These findings indicate that BAL therapy has a protective effect on renal function and mitigates renal failure. In summary, HepGLs-BAL therapy effectively alleviated inflammatory factor levels, reduced the release of endotoxins, and consequently mitigated kidney injury.

### BAL therapy alleviates hepatic encephalopathy

**Figure 7:**
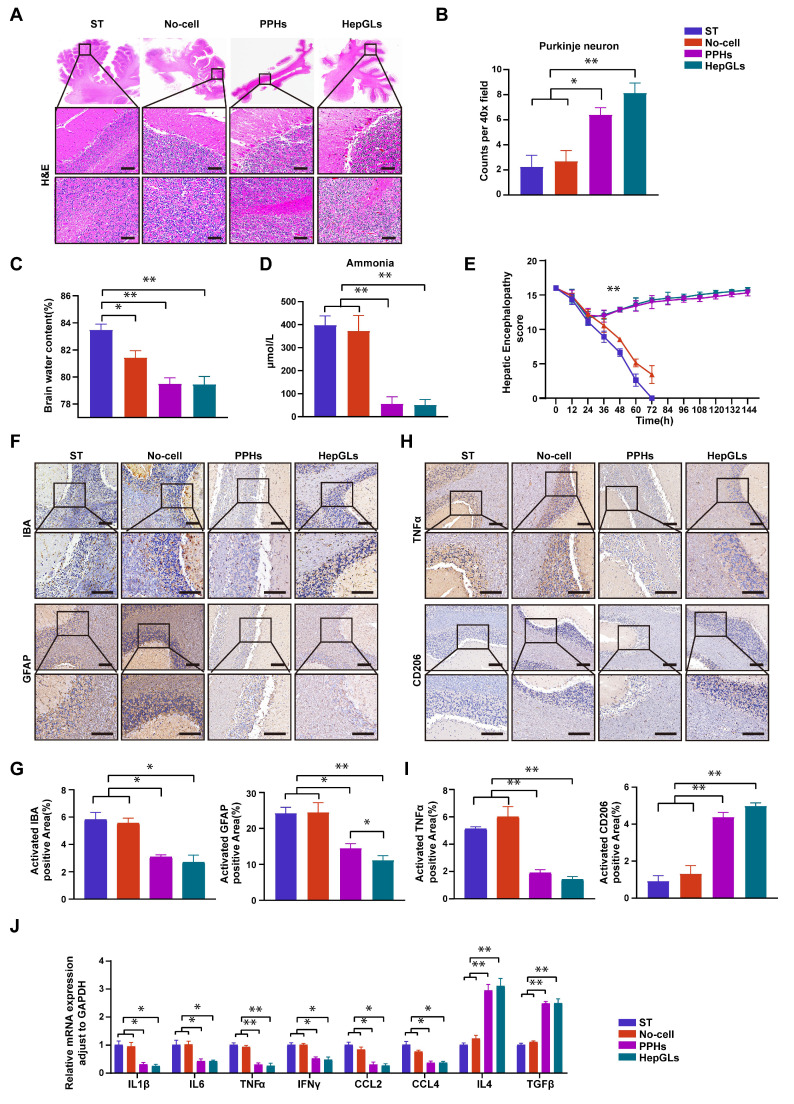
** BAL therapy alleviates hepatic encephalopathy.** (A) H&E staining of cerebellum tissue from the ST, No-cell, PPHs and HepGLs groups at the endpoint (Scale bars=100μm; n=5 per group). (B)Quantification of purkinje neuron. (C)Brain water content in the four groups at the endpoint (n=5 per group). (D) Plasma ammonia in the four groups during the experiment (n=5 per group). (E) HE scores in the four groups during the experiment (n=5 per group). (F) Immunohistochemistry staining of cerebellum for IBA and GFAP in the four groups (Scale bars=100μm; n=5 per group). (G) Quantification of (F). (H) Immunohistochemistry staining of cerebellum for TNFα and CD206 in the four groups (Scale bars=100μm; n=5 per group). (I) Quantification of (H). (J) Real-time PCR analysis of mRNA levels of pro-inflammatory genes in cerebellum samples from the four groups at the endpoint. The data represent the mean ± SEM. Statistical significance was assessed by two-tailed Student's t-test and two-way ANOVA. *p < 0.05, **p < 0.01, and ***p < 0.001; n.s., not significant.

Hepatic encephalopathy encompasses a range of neuropsychiatric changes that manifest in individuals with liver failure^22^. The brain tissue alterations associated with hepatic encephalopathy are diverse and include cerebral edema, imbalances in neurotransmitter levels, neuronal damage, and an inflammatory response^23^. These changes contribute to neurosystemic dysfunction and cognitive deterioration in patients affected by hepatic encephalopathy^24^. H&E staining revealed significant loss of Purkinje neurons and increased brain water content in the ST and No-cell groups, indicating the occurrence of neuronal damage and brain edema (Fig. 7A-C). The levels of ammonia, a crucial factor for evaluating the onset and progression of hepatic encephalopathy, were substantially greater in the ST and no-cell groups than in the two BAL groups (Fig. 7D). The HE score was used to assess the severity of nerve injury, which decreased in all groups following D-gal infusion. Notably, the score increased significantly after 8 h of treatment and gradually returned to normal levels by the study endpoint in the two BAL groups (Fig. 7E).

Microglia and astrocytes play a role in regulating brain homeostasis and modulating inflammation, becoming activated when neural dysfunction occurs^25^. Compared with those in the two BAL groups, the expression of IBA-1 and GFAP, which are markers of microglia and astrocytes, respectively, was greater in the ST and No-cell groups (Fig. 7F-G). Microglia can polarize to one of two distinct activation states, known as classic activation (M1 phenotype) and alternative activation (M2 phenotype), a phenomenon referred to as polarization^26^. M1 microglia are considered proinflammatory cells and release elevated levels of proinflammatory factors such as IFN-γ, TNF-α, and IL-1β upon activation. In contrast, the M2 phenotype exerts an opposing effect by promoting the release of anti-inflammatory factors. TNFα and CD206 levels were utilized to assess the cellular state, and the results revealed a significant decrease in TNF-α levels in the two BAL groups. Conversely, the expression of CD206 was notably greater in the two BAL groups than in the ST and no-cell groups (Fig. 7H-I).

To evaluate the overall inflammatory status of the brain, the mRNA expression of several proinflammatory and anti-inflammatory genes was assessed. The results revealed a significant decrease in the expression of proinflammatory genes, including IL-1β, IL-6, TNFα, IFN-γ, CCL2, and CCL4, in the two BAL groups. Conversely, the expression of anti-inflammatory genes (IL4 and TGF-β) markedly increased compared with that in the ST and No-cell groups (Fig. 7J). Taken together, these findings suggest that HepGL-BAL therapy can confer brain-protective effects by mitigating cerebral edema and reducing inflammation.

## Discussion

ALF is characterized by severe and massive hepatocyte injury, resulting in liver function impairment and multi-organ failure^27^. BAL therapy aims to metabolize toxins associated with liver failure, improve the internal environment, suppress inflammatory factor storms, and promote liver regeneration^28^, ^29^. The effectiveness of BAL therapy relies on sufficient functional hepatocytes and compatible bioreactors, which are the major challenges for BAL clinical translation. Although human hepatocytes are widely regarded as the clinical standard for BAL applications, their use is often limited by challenges in obtaining a sufficient and functional supply due to restricted availability and phenotypic variability^7^. Inducing the differentiation of human-derived stem cells into hepatocytes is a promising approach for obtaining seed cells^30^. However, the efficiency and long-term stability of these methods still lack relevant scientific evidence. Here, we revealed that CCL2 can effectively support the long-term expansion of human hepatocytes *in vitro* while retaining specific liver functions. HepGLs can repopulate and restore the livers of Fah^⁻/⁻^ mice following NTBC withdrawal, effectively alleviating ALF. Furthermore, we demonstrated that HepGLs-BAL can effectively mitigate ALF and extrahepatic organ failure in porcine preclinical models.

Hepatocyte dedifferentiation to acquire proliferative capacity, recognized as a crucial mechanism for liver self-repair in response to injury, is regulated by the Hippo signaling pathway. Researchers have reported that TNFα and Wnt3 may enhance this process^31^. A combination of small molecule compounds can promote human primary hepatocyte dedifferentiation and the acquisition of proliferative capacity *in vitro*, making them potentially effective cell sources for BAL therapy. However, it remains unclear whether there is a more efficient and simple method to support the clonal expansion of human hepatocytes. Researchers have demonstrated that CCL2 expression is highly upregulated in the livers of ALF patients and ALF mice and that mesenchymal stem cells overexpressing CCR2 can specifically target damaged livers and effectively alleviate liver injury^32^. In our study, we employed single-cell transcriptomic analysis and confirmed that CCL2 expression is upregulated in ALF patients and is closely associated with the expression of genes involved in liver regeneration and differentiation. Using a previously described hepatic maturation protocol, subsequent *in vitro* experiments revealed that CCL2 expression can reprogram human hepatocytes for long-term expansion^4^. HepGLs exhibit gene expression profiles and functional characteristics similar to those of hepatocytes and can largely repopulate the livers of Fah^⁻/⁻^ mice, alleviating ALF. This efficient method to expand human hepatocytes *in vitro* may have potential applications in liver regeneration therapy.

In the D-gal-induced porcine ALF experiment, BAL therapy with HepGLs and PPHs significantly improved the survival rate. We observed the occurrence of hepatic encephalopathy in the control and no-cell groups, whereas neurological signs significantly improved or almost disappeared in the BAL groups. Additionally, the blood ammonia levels in the BAL group were significantly lower than those in the control group, further indicating the ammonia removal capability of BAL therapy. During the treatment period, the urea concentration in the bioreactor gradually increased, confirming that the PPHs and HepGLs cells were able to detoxify ammonia via ureagenesis. Previous studies have reported that neuroinflammation mediates cognitive impairment and worsens hepatic encephalopathy^33^. In our study, pathological analysis revealed a significant decrease in the number of M1-type cells and a significant increase in the number of M2-type microglia after BAL therapy, further indicating the mitigating effect of BAL therapy on neuroinflammation.

Sustained hepatocyte regeneration plays a vital role in ALF recovery^34^. Immunohistochemical analysis revealed a significant increase in the number of Ki67-positive cells after BAL therapy, confirming the stimulation of hepatocyte regeneration. Wei-Jian Li et al proposed that FOXA3-induced LPCs may promote the secretion of multiple human growth factors, including HGF and TGF-α, thus resolving toxic reactions and promoting liver recovery^5^. In our study, we observed a significant increase in the levels of liver regeneration-related factors after HepGLs-BAL therapy. This effect implies that HepGLs can secrete human growth factors, thereby tipping the balance toward the resolution of toxic reactions and facilitating early-stage liver regeneration. Liver dysfunction induced by D-gal primarily occurs within the first 24 h, followed by an inflammatory storm that exacerbates liver injury. Inflammatory cytokines may further aggravate liver damage through the induction of inflammatory cascades. In our study, after HepGLs-BAL therapy, the levels of porcine plasma inflammatory cytokines such as IFN-γ, IL-1 and IL-6 were significantly reduced. HepGLs-BAL therapy may impede the progression of inflammation, diminish apoptosis, and thus improve liver damage in ALF, as validated by histopathological analysis at various time points. BAL therapy effectively alleviates and slows the progression of inflammation, thereby mitigating liver injury and providing a favorable environment for liver recovery, which increased the survival rate of the animals.

Intestinal mucosal barrier dysfunction and increased intestinal permeability can result in bacterial translocation and thus trigger an inflammatory response, ultimately leading to the development of systemic inflammatory response syndrome (SIRS) and multiple organ dysfunction syndrome (MODS)^35^. Additionally, intestinal *E. coli* and endotoxins can penetrate the intestinal epithelial barrier, enter the bloodstream, and stimulate the mononuclear macrophage system, causing the release of many cytotoxic factors^19^. This further exacerbates the systemic inflammatory response, leading to the development of AKI. The incidence of AKI in patients with ALF reaches 38-70%, and it is associated with a mortality rate of 20%^36^. BAL therapy improved the integrity of the intestinal mucosal barrier, effectively reduced plasma endotoxin levels and decreased *E. coli* levels in the liver and kidney. Consequently, plasma creatinine levels significantly decreased following BAL treatment. Taken together, these findings confirm that BAL therapy can attenuate the immune response and mitigate damage to the gut-liver axis, consequently improving ALF and its associated complications.

This study presents research on BAL therapy for ALF in miniature pigs based on HepGLs. These findings confirm the safety and efficacy of BAL therapy, as it effectively mitigates liver injury, reduces serum ammonia levels and promotes liver regeneration. However, this study has several limitations. During our treatment, the AST and ALT levels in the animals were more than 100 times higher than the normal levels, indicating severe liver damage. This could not accurately simulate the treatment before severe liver dysfunction in clinical human patients. Additionally, D-Gal is a hepatocyte-specific toxin that closely resembles APAP-induced human liver failure, but it may fail to fully simulate ACLF caused by HBV, HCV, or NASH. Further clinical trials are necessary to examine the efficacy of BAL therapy and determine its potential benefits for ALF patients.

## Conclusion

In summary, we successfully utilized CCL2 to induce the expansion of primary human hepatocytes and confirmed that HepGLs-BAL therapy may effectively rescue miniature pigs with ALF. HepGLs-BAL therapy effectively reduces serum ammonia levels, promotes liver regeneration, suppresses inflammatory responses, protects extrahepatic organs, and improves the survival rate of ALF pigs. These findings provide a potential new direction for the clinical treatment of ALF.

## Supplementary Material

Supplementary methods, figures and tables.

## Figures and Tables

**Table 1:** Treatment parameters and outcomes

Group		Weight(kg)	Cell number (10^9^)	Duration (h)	NH3 (endpoint) μmol/L	Survival time (h)
Control	35.1		0	0	514	48
Control	37		0	0	405	60
Control	36.5		0	0	453	60
Control	38		0	0	326	52
Control	40.1		0	0	138	68
No-cell	36.5		0	8	481	48
No-cell	37.2		0	8	376	54
No-cell	41.3		0	8	261	60
No-cell	35.5		0	8	281	68
No-cell	33.4		0	8	131	76
PPHs	32.3		3.5	8	57	Survived
PPHs	37		2.9	8	76	98
PPHs	39		4.1	8	90	Survived
PPHs	36.5		4.3	8	86	Survived
PPHs	40.5		3.8	8	55	Survived
HepGLs	40		3.6	8	52	Survived
HepGLs	38		4.0	8	54	Survived
HepGLs	35.5		3.3	8	47	Survived
HepGLs	37.2		4.8	8	61	Survived
HepGLs	36		4.7	8	35	Survived
						
