# Supplementary material for: CCL2 supports human hepatocytes long-term expansion for bioartificial liver therapy to relieve acute liver failure and extrahepatic complications
Source: Int J Biol Sci. 2025 Oct 20;21(15):6759–74. doi: 10.7150/ijbs.115293 (PMC12631109; doi:10.7150/ijbs.115293)
Supplement: Supplementary file 1 — Supplementary methods, figures and tables. [file ijbsv21p6759s1.pdf]

Supplementary material

CCL2 supports human hepatocytes long-term expansion for bioartificial liver therapy to relieve acute liver failure and extrahepatic complications

Materials and Methods

Hepatocyte culture and ammonia elimination examination

For this study, we obtained human hepatocytes from the normal liver tissues of four individuals who underwent liver resection surgery at Zhujiang Hospital (Southern Medical University, Guangzhou, China) due to hepatic hemangioma. The isolation and culture of human hepatocytes were adapted from a previously reported method. Briefly, the liver tissues were subjected to type I collagenase digestion, followed by centrifugation, filtration, and other steps until human primary hepatocytes were obtained. The hepatocytes were cultured at 37°C in an incubator with 5% CO2 in air atmosphere, and expanded to 10^8 cells. On day 9 of the primary culture, cells were trypsinized and then seeded at a density of $1x10^4$ cells/cm2. The medium was changed every 2 days and were frozen in serum-free cell cryopreservation solution through CELLSAVING^{TM} (C40100, New Cell & Molecular Biotech). HepGLs were cultured for about 9 days per passage, with passages 5 through 10 being used for BAL therapy. Ammonia elimination was assessed by incubating hepatocyte with 3 mM NH4Cl in HepatoZYME-SFM. The concentration of ammonia in the supernatant collected 24 hours after induction was measured using enzymatic colorimetric assays from Megazyme International.

Large-scale amplification of HepGLs in the bioreactor

The bioreactor was designed to ensure efficient oxygen exchange, adequate nutrient supply, and a three-dimensional (3D) cultivation environment for hepatocytes. At the center of the bioreactor was a fixed bed composed of fiber scaffolds made from medical-grade polyester microfibers (11.3 cm^2 per unit), providing a maximum growth surface area of 4 m^2 (Fig. 3A). HepGLs were initially seeded into plate culture bottles, expanded to 10^8 cells, and subsequently transferred to the bioreactor culture system. Since the rate of blood glucose reduction is directly correlated with the cell count in the bioreactor, it is essential to replace the culture medium regularly based on the glucose concentration in the circulating medium (i.e., when it drops

below 3 mmol/L or after 5 days of culture). This process is continued until the HepGLs reach an amplified range of 10^9 to 10^{10} cells.

Animals

All mice were housed in a controlled light-dark cycle of 12 hours each. We complied with all relevant ethical regulations with approved study protocols. All experiments were approved by the Animal Care and Use Committee of Zhujiang Hospital, Southern Medical University (LAEC-2022-236).

Tibetan miniature pigs weighing between 30-45 kg and aged 1-2 years, were purchased from Guangdong Pearl Biotechnology Co., Ltd. The animals were randomly divided into four groups: supportive therapy (ST, n = 5), BAL without cells (No-cell, n = 5), BAL with PPHs (PPHs, n = 5), and BAL with HepGLs (HepGLs, n = 5). Fah-KO(M-NSG) mice (Cat. NO. NM-NSG-004) were purchased from Shanghai Model Organisms Center, Inc. All animals underwent adaptive feeding for 5 days and then were fasted for 12 hours before experimentation. The animal experiments were approved by the Animal Care and Use Committee of Zhujiang Hospital, Southern Medical University (LACE-2023-056).

Immunofluorescence staining, PAS staining and ICG assay

Liver tissues ware fixed in formalin for 24 hours and subsequently embedded in paraffin. Section of 4μm thickness were prepared for immunohistochemistry (IHC) and immunofluorescence staining. Briefly, after undergoing heat-induced epitope retrieval, the paraffin-embedded sections were treated with 3% H_2O_2 and blocked with a 5% bovine serum albumin buffer. Primary antibodies were incubated overnight at 4°C, followed by incubation with secondary antibody for 1 hour at room temperature. For IHC staining, the DAB Peroxidase Substrate Kit (DAB; DA1010; Solarbio, China) was used for visualization, and cell nuclei were counterstained with hematoxylin. Tissue sections were observed using brightfield microscopy. For immunofluorescence, sections were counterstained with DAPI for nuclear visualization. Images of stained cells were captured using a fluorescence microscope.

To assess the glucose storage ability of hepatocytes, cells were fixed with 4% PFA and stained using PAS kit (Sigma, USA) in accordance with the manufacturer’s instructions. To analyze the uptake capacity of hepatocytes for ICG, the culture medium was replaced with fresh

medium containing 1 mg/ml ICG and incubated for 1 hour. The cells were then fixed with 4% PFA for 30 minutes. Images were captured with a fluorescence microscope.

Porcine drug-induced ALF model establishment and therapy

The experimental animals were administered anesthesia with 30 mg/kg of sodium pentobarbital and 0.1 ml/kg of Xylazine hydrochloride injection. Once the anesthesia took effect, double-lumen hemodialysis catheters were inserted into the jugular vein and femoral vein respectively. These catheters were utilized for blood collection, maintaining anesthesia and BAL treatment. According to previous studies, a dose of 0.45g/kg D-gal was infused through the jugular vein to construct a liver failure model. The animals were then allowed to recover, and their clinical vital signs were monitored. Blood samples were collected every 12 hours. After 24 hours of D-gal injection, the experimental animals were connected to a combined artificial liver system via the jugular vein and femoral vein and underwent 8 hours of treatment. During the therapy, blood samples were collected every 2 hours for chemical analysis. The blood biochemical test indicators included albumin (ALB), aspartate aminotransferase (AST), alanine aminotransferase (ALT), blood urea nitrogen (BUN), total bilirubin (TBIL), creatinine (Cr), glucose, ammonia and coagulation function testing (DRI-7000i, FUJI and URIT). All samples were stored at -80 °C for further analysis. The experimental timeline is presented in Figure 3M. The study endpoint was defined as either animal death or survival for 144 hours.

Biochemical assays and endotoxin

All blood samples were collected and centrifuged at 4000 rpm for 10 minutes to obtain plasma. The plasma chemicals were assessed using biochemical instruments (DRI-7000i, FUJI and URIT). Plasma endotoxin concentrations were measured using the Endotoxin Detection Kit (B50-600L; LONZA) according to the manufacturer’s instructions.

Reverse transcription-quantitative PCR (RT-qPCR)

Total RNA was extracted from liver and brain tissues or cells using TRIzol Reagent (Life Technologies, USA) following the manufacturer’s instructions. RNA samples were reverse transcribed to cDNA using the Evo M-MLV RT Kit with gDNA Clean for qPCR (AG11711, ACCURATE BIOTECHNOLOGY(HUNAN） CO.,LTD, ChangSha, China). Subsequently, qPCR was performed using 2X SYBR Green Pro Taq HS Premix IV (AG11746, ACCURATE

BIOTECHNOLOGY(HUNAN) CO.,LTD, ChangSha, China). All qPCR data were repeated three times, and the results were normalized to GAPDH expression.

Protein assays

AKI-associated plasma proteins were assessed using an AKI antibody array (ab169806; Abcam) according to the manufacturer's instructions. Protein from organoids and cells were visualized using EZ ECL pico luminescence reagent (AP34L025, Life-iLab, Shanghai, China) on a ChemiDoc MP Imaging System (Bio-Rad; Hercules, CA, USA). Plasma cytokines were assessed using the Proteome Profiler Cytokine Array Kit (ARY022B; R&D systems) containing 105 different cytokine antibodies, also according to the manufacturer's instructions.

Transmission electron microscopy (TEM)

The kidney tissue sections were initially fixed using a 2.5% glutaraldehyde solution, followed by post-fixation with 1% osmium tetroxide (OsO_4) for 2 hours on ice. Subsequently, the tissues were washed with PBS, dehydrated using ethanol and propylene oxide, and finally embedded in an Epon mixture. The polymerized blocks yielded were sectioned, mounted on grids, stained with uranyl acetate and lead citrate, and examined using HITACHI HT7800 transmission electron microscopy. To analyze the subcellular structures, at least 10 images were captured for each sample.

Single-cell RNA-seq and data analysis

Publicly available scRNA-seq data from human fetal liver development was utilized in this study. The single-cell RNA sequencing datasets were cited from "Single-cell atlas of human liver development reveals pathways directing hepatic cell fates" and downloaded from ArrayExpress under accession code E-MTAB-7407 and E-MTAB-8210. Quality control, dimensionality reduction and unsupervised clustering of the scRNA-seq data were performed using the 'Seurat' package. Each sample was individually assessed for quality, and cells were filtered based on gene coverage, read counts and mitochondrial content. Cells expressing more than 15% mitochondrial content were excluded. Dimensionality reduction, clustering and cluster biomarkers were performed using the Seurat package in R. Non-linear dimensional reduction was conducted using the UMAP method. Differential gene expression was visualized using a volcano plot generated in GraphPad Prism 8.0 software. All analyses in the manuscript were performed using R software (version 4.3.2).

Bulk transcriptome data

Bulk transcriptome data were obtained from the GEO database (ID: GSE4528, GSE38941, GSE74000, and GSE120652). The "AnnoProbe" R package was used to map the probes. The "limma" R package was employed to calculate the average values of multiple probes. The "combat" function from the "sva" R package was employed in these datasets.

Organoid generation

HepGLs cells were harvested as single cells and then placed into Matrigel embeds (Corning, 354248) to induce hepatic endoderm spheroid differentiation. Subsequently, they were seeded into a 6-well ultra-low attachment plate (Corning, 3471) at a density of 7.5^105 cells per well. The culture medium consisted of HepatoZYME-SFM without EGF, diluted with Endothelial Cell Growth Medium-2 (Lonza, CC 3162) in a 1:1 ratio and supplemented with 2.5% FBS, 100 nM dexamethasone, 20 ng/ml OSM (MCE, HYP70465), and 10 ng/ml of HGF (MCE, HY-P70627). The culture medium was changed every two days.

Imaging functional bile canaliculi

The organoids were subjected to standard preparation. This involved a single wash with DPBS Ca/Mg (Gibco, 14040133) followed by Hoechst 33342 staining in HepatoZYME-SFM (Gibco, 17705021) for 30 minutes at 37°C. For snapshot imaging, organoids were incubated in HepatoZYME-SFM medium supplemented with 1 mmol/L CDFDA (Thermo Fisher, F1303) for 2 hours at 37°C. After washing twice with DPBS Ca/Mg, the organoids were transferred to a well of an m-slide Angiogenesis glass-bottom plate (Ibidi, Bayern, Germany) filled with HepatoZYME-SFM. For time-lapse imaging, the organoids were continuously cultured in HepatoZYMESFM supplemented with 1 mmol/L CDFDA.

Statistical analysis

Quantitative data (mean ± standard deviation) were subjected to either the student t-test or ordinary one-way analysis of variance (ANOVA) multiple comparison test, depending on the experimental design. A value of $P<0.05$, 0.01, or 0.001 was considered statistically significant. GraphPad Prism 9 software (GraphPad Software, SanDiego, CA) was used for these statistical analyses.

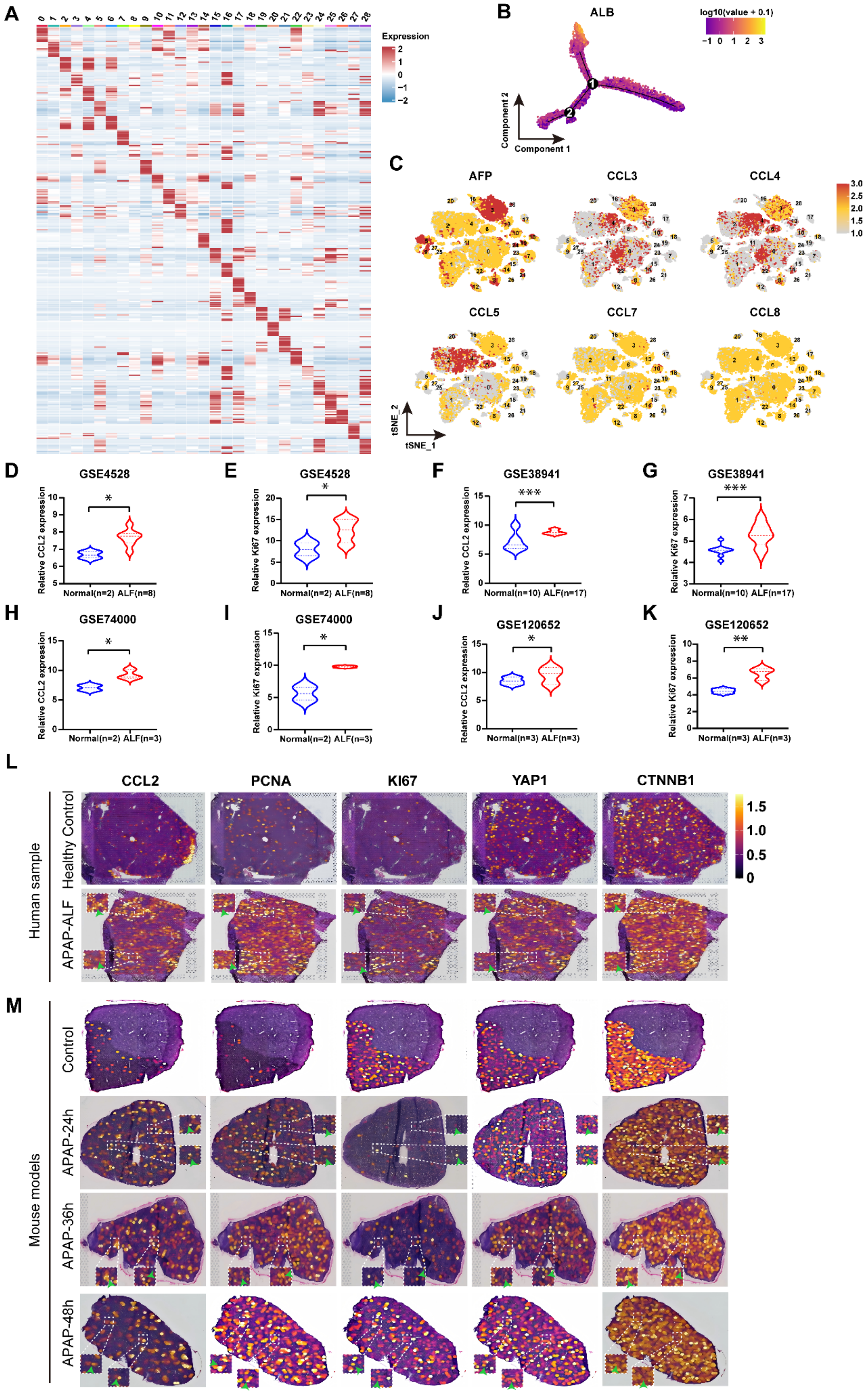
A
Expression
B
ALB
log10(value + 0.1)
Component 2
Component 1
C
AFP
CCL3
CCL4
CCL5
CCL7
CCL8
tSNE_2
tSNE_1
D
GSE4528
Relative CCL2 expression
Normal(n=2) ALF(n=8)
E
GSE4528
Relative Ki67 expression
Normal(n=2) ALF(n=8)
F
GSE38941
Relative CCL2 expression
Normal(n=10) ALF(n=17)
G
GSE38941
Relative Ki67 expression
Normal(n=10) ALF(n=17)
H
GSE74000
Relative CCL2 expression
Normal(n=2) ALF(n=3)
I
GSE74000
Relative Ki67 expression
Normal(n=2) ALF(n=3)
J
GSE120652
Relative CCL2 expression
Normal(n=3) ALF(n=3)
K
GSE120652
Relative Ki67 expression
Normal(n=3) ALF(n=3)
L
CCL2
PCNA
KI67
YAP1
CTNNB1
Human sample
Healthy Control
APAP-ALF
M
Mouse models
Control
APAP-24h
APAP-36h
APAP-48h

Figure S1. CCL2 is upregulated in ALF patients and mice.

(A) Heatmap showing the expression of marker genes in the indicated cell types. (B) Pseudotime analysis revealed the ALB expression during development. (C) Feature plots of specific marker genes. (D-K) Gene expression analysis in liver tissues from patients and mice with ALF using GEO datasets from NCBI database. (L) Gene expression analysis in liver tissues from patients with APAP-induced ALF using spatial transcriptome data. (M) Gene expression analysis in liver tissues from mice with APAP-induced ALF using spatial transcriptome data. The data represent the mean ± SEM. Statistical significance was assessed by two-tailed Student's t-test and two-way ANOVA. $^*p < 0.05$, $^{**}p < 0.01$, and $^{***}p < 0.001$; n.s., not significant.

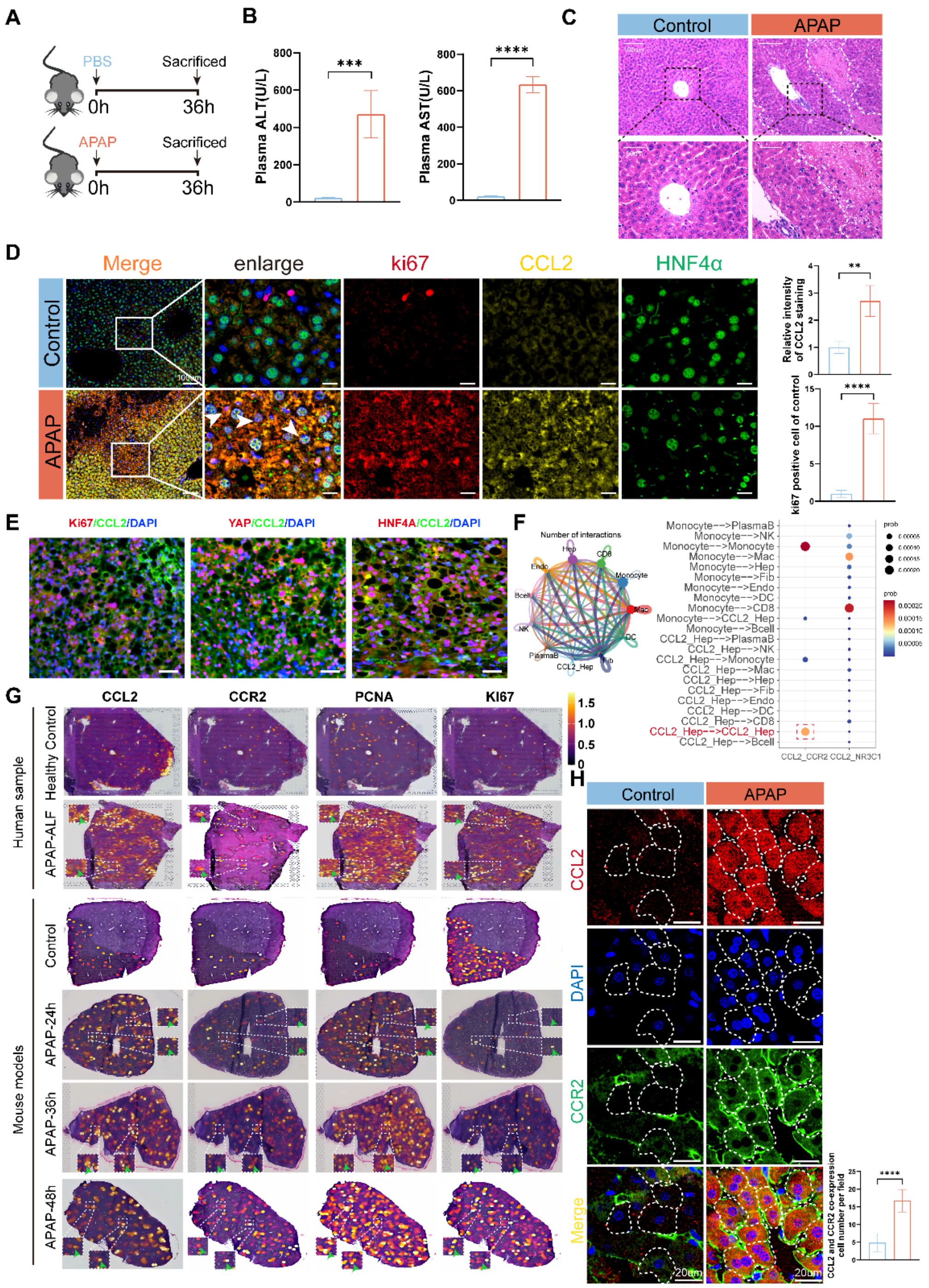
A
PBS
Sacrificed
0h
36h
APAP
Sacrificed
0h
36h
B
Plasma ALT(U/L)
***
Plasma AST(U/L)
****
C
Control
APAP
D
Merge
enlarge
ki67
CCL2
HNF4α
Control
APAP
Relative intensity of CCL2 staining
**
ki67 positive cell of control
****
E
Ki67/CCL2/DAPI
YAP/CCL2/DAPI
HNF4A/CCL2/DAPI
F
Number of interactions
Hep
CD8
Endo
Monocyte
Bcell
Mac
NK
DC
PlasmaB
CCL2_Hep
Fib
Monocyte-->PlasmaB
Monocyte-->NK
Monocyte-->Monocyte
Monocyte-->Mac
Monocyte-->Hep
Monocyte-->Fib
Monocyte-->Endo
Monocyte-->DC
Monocyte-->CD8
Monocyte-->CCL2_Hep
Monocyte-->Bcell
CCL2_Hep-->PlasmaB
CCL2_Hep-->NK
CCL2_Hep-->Monocyte
CCL2_Hep-->Mac
CCL2_Hep-->Hep
CCL2_Hep-->Fib
CCL2_Hep-->Endo
CCL2_Hep-->DC
CCL2_Hep-->CD8
CCL2_Hep-->CCL2_Hep
CCL2_Hep-->Bcell
CCL2_CCR2 CCL2_NR3C1
prob
G
CCL2
CCR2
PCNA
KI67
Human sample
Healthy Control
APAP-ALF
Mouse models
Control
APAP-24h
APAP-36h
APAP-48h
1.5
1.0
0.5
0
H
Control
APAP
CCL2
DAPI
CCR2
Merge
20um
CCL2 and CCR2 co-expression cell number per field
****

Figure S2. CCL2 promotes hepatocyte regeneration through an autocrine CCL2-CCR2 loop.

(A) Schematic diagram of the APAP-induced acute liver failure (APAP-ALF) model in 8-week-old C57BL/6J mice (n=5 per group). (B) Serum AST and ALT levels in control and APAP-ALF mice (n=5 per group). (C) Representative H&E staining of liver sections from APAP-ALF mice (scale bars, 200 μm and 100 μm). (D) Immunofluorescence staining of CCL2, HNF4α, and Ki67 with quantification in liver tissues from control and APAP-ALF mice (scale bar, 100 μm). (E) Immunofluorescence images of CCL2, Ki67, YAP and HNF4A in APAP-induced ALF recovery human liver (Scale bars, 100μm). (F) Ligand–receptor interaction analysis showing ligands expressed by hepatocytes and cognate receptors on immune cells. Red highlights indicate robust interactions, suggesting that CCL2-high hepatocytes bind to CCR2 via autocrine signaling. (G) Spatial transcriptomic analysis of CCL2, CCR2, Ki67, and PCNA expression in human and mouse livers with APAP-induced ALF. Green arrows indicate co-localization of the indicated genes. (H) Immunofluorescence staining showing CCR2 (green) and CCL2 (red) in liver sections from control and APAP-ALF mice; nuclei were counterstained with DAPI (scale bar, 20 μm). Quantification of CCR2 and CCL2 co-expression in liver sections from control and APAP-ALF mice. The data represent the mean ± SEM. Statistical significance was assessed by two-tailed Student's t-test and two-way ANOVA. *$p < 0.05$, **$p < 0.01$, and ***$p < 0.001$; n.s., not significant.

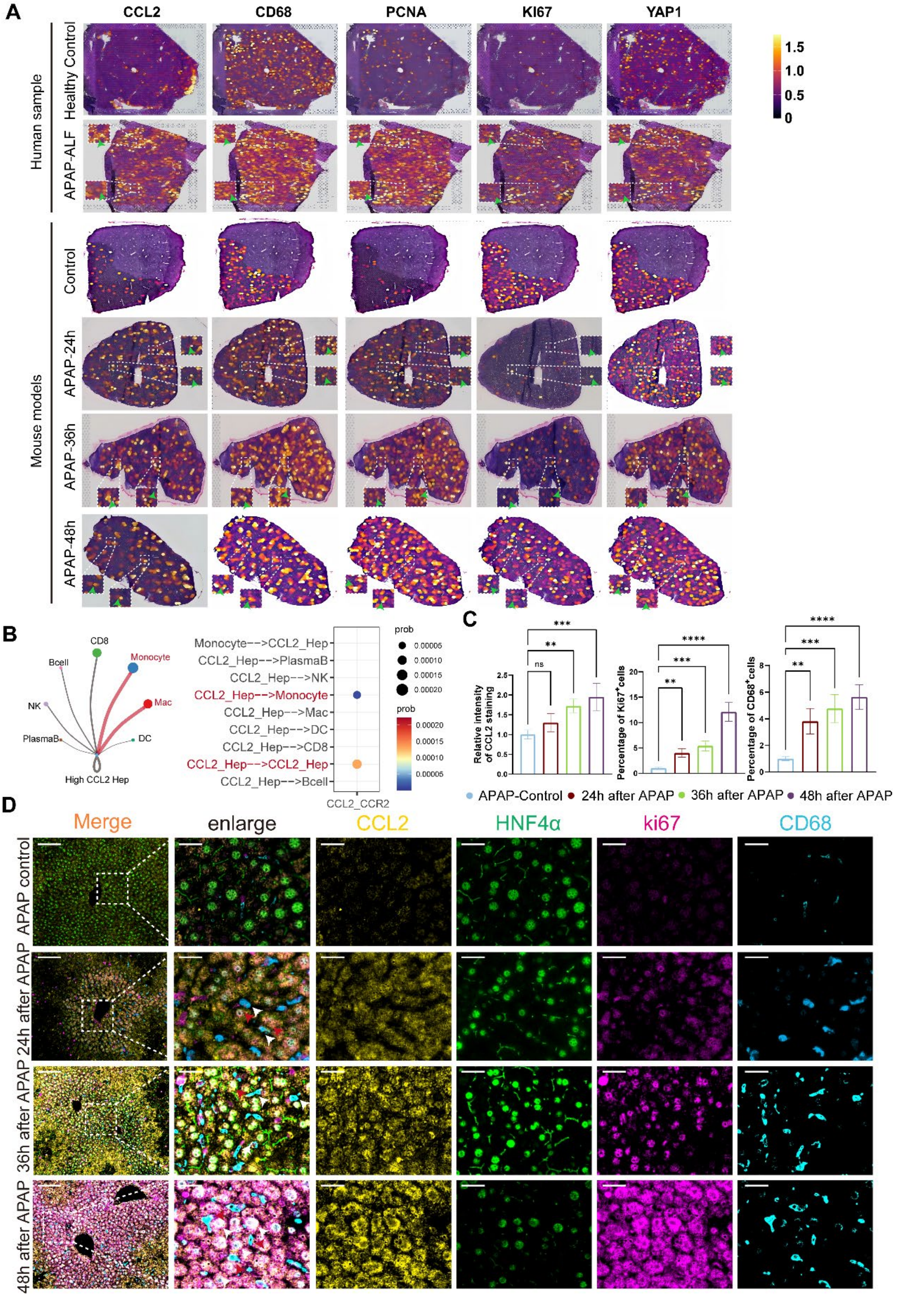

A
CCL2
CD68
PCNA
KI67
YAP1
Human sample
Healthy Control
APAP-ALF
Mouse models
Control
APAP-24h
APAP-36h
APAP-48h
1.5
1.0
0.5
0
B
CD8
Monocyte
Bcell
Mac
NK
DC
PlasmaB
High CCL2 Hep
Monocyte-->CCL2_Hep
CCL2_Hep-->PlasmaB
CCL2_Hep-->NK
CCL2_Hep-->Monocyte
CCL2_Hep-->Mac
CCL2_Hep-->DC
CCL2_Hep-->CD8
CCL2_Hep-->CCL2_Hep
CCL2_Hep-->Bcell
CCL2_CCR2
prob
0.00005
0.00010
0.00015
0.00020
C
Relative intensity of CCL2 staining
Percentage of Ki67+cells
Percentage of CD68+cells
ns
**
***
****
APAP-Control
24h after APAP
36h after APAP
48h after APAP
D
Merge
enlarge
CCL2
HNF4α
ki67
CD68
APAP control
24h after APAP
36h after APAP
48h after APAP

Figure S3. Interactions between CCL2-high hepatocytes and myeloid cells during liver regeneration.

(A) Spatial transcriptomic analysis of CCL2, CD68, Ki67, PCNA, and YAP1 expression in liver tissues from human and mouse APAP-induced ALF samples. (B) Ligand–receptor interaction analysis showing ligands expressed by hepatocytes and cognate receptors on macrophages/monocytes. Red highlights indicate robust interactions between CCL2-high hepatocytes and myeloid cells. (C–D) Representative immunofluorescence images and quantification of CCL2, HNF4α, CD68, and Ki67 expression in liver tissues from control and APAP-induced ALF mice at 24, 36, and 48 hours post-administration (n=5 per group, scale bar, 100 μm). The data represent the mean ± SEM. Statistical significance was assessed by two-tailed Student's t-test and two-way ANOVA. $^{*}p < 0.05$, $^{**}p < 0.01$, and $^{***}p < 0.001$; n.s., not significant.

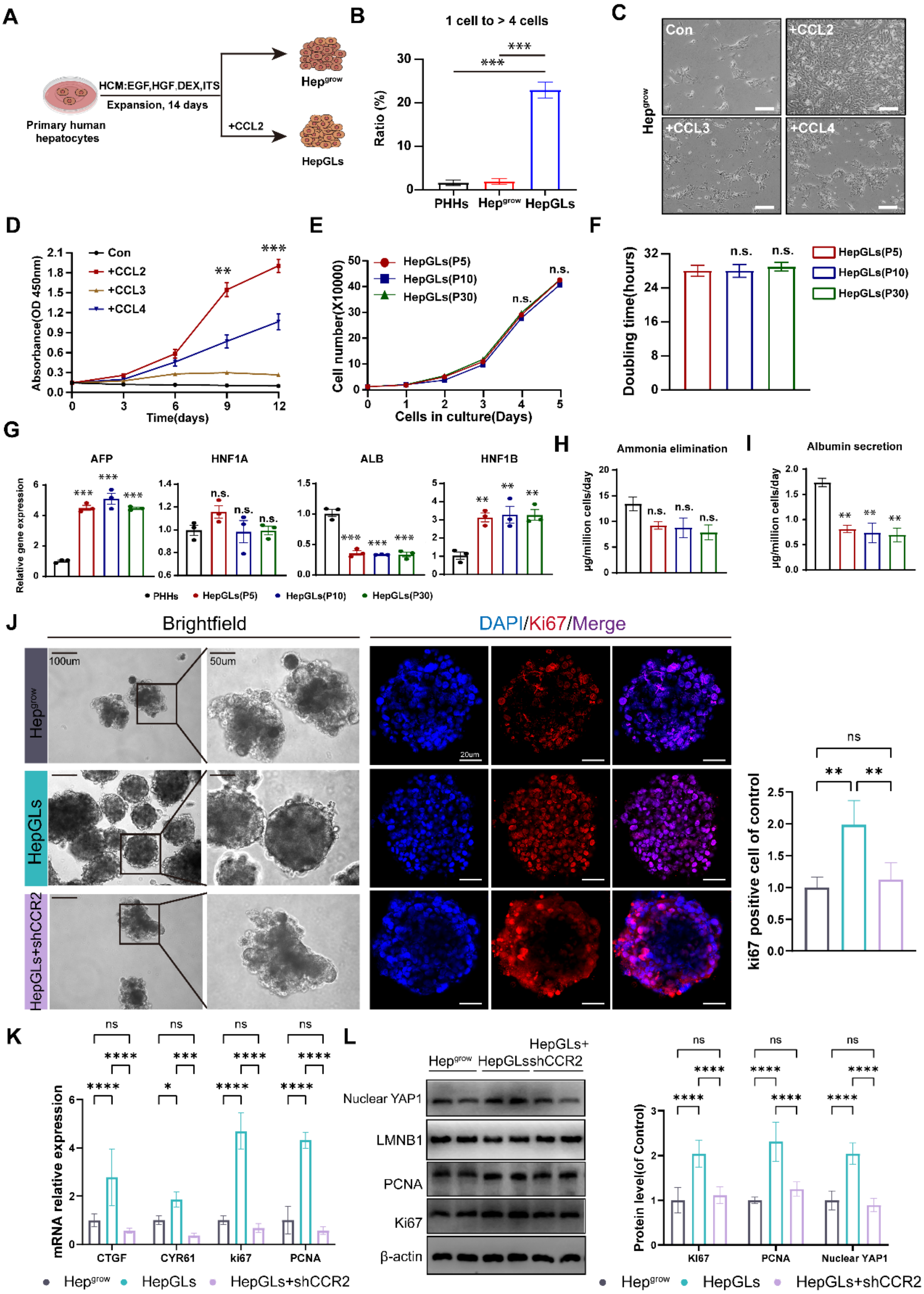

A
HCM:EGF,HGF,DEX,ITS
Expansion, 14 days
Primary human hepatocytes
+CCL2
Hepgrow
HepGLs
B
1 cell to > 4 cells
Ratio (%)
PHHs
Hepgrow
HepGLs
C
Con
+CCL2
+CCL3
+CCL4
Hepgrow
D
Absorbance(OD 450nm)
Time(days)
Con
+CCL2
+CCL3
+CCL4
E
Cell number(X10000)
Cells in culture(Days)
HepGLs(P5)
HepGLs(P10)
HepGLs(P30)
F
Doubling time(hours)
G
AFP
HNF1A
ALB
HNF1B
Relative gene expression
PHHs
HepGLs(P5)
HepGLs(P10)
HepGLs(P30)
H
Ammonia elimination
μg/million cells/day
I
Albumin secretion
μg/million cells/day
J
Brightfield
DAPI/Ki67/Merge
100um
50um
20um
Hepgrow
HepGLs
HepGLs+shCCR2
ki67 positive cell of control
K
mRNA relative expression
CTGF
CYR61
ki67
PCNA
Hepgrow
HepGLs
HepGLs+shCCR2
L
HepGLs+
Hepgrow
HepGLs
shCCR2
Nuclear YAP1
LMNB1
PCNA
Ki67
β-actin
Protein level(of Control)
KI67
PCNA
Nuclear YAP1

Figure S4. CCL2 promotes human primary hepatocyte expansion in vitro

(A) Schematic diagram of primary human hepatocytes expansion using a chemical cocktail and CCL2. (B) The frequency of single cells that produced more than four cells at the end of time-lapse imaging (3 days). (C) Representative images of the Hep^{grow} cultured with CCL2, CCL3, or CCL4 (Scale bars=100μm). (D) Absorbance of the Hep^{grow} cultured with CCL2, CCL3, or CCL4 for 12 days (n=5 per group). (E) Growth curves of HepGLs at passages P5, P10 and P30 (n=3 per group). (F) Doubling time of HepGLs at passages P5, P10 and P30 (n=3 per group). (G) Real-time PCR analysis of mRNA levels of hepatocyte functional genes in PHHs and HepGLs at passages P5, P10 and P30 (n=3 per group). (H-I) Ammonia elimination and Albumin secretion of PHHs and HepGLs at passages P5, P10 and P30 (n=3 per group). (J) Bright-field images of HepGLs organoids cultured with exogenous CCL2 or with CCR2 knockdown (scale bar, 100 μm, n=5 per group). Representative Ki67 immunofluorescence images with quantification are shown (scale bar, 20 μm). (K) qPCR analysis of Ki67, PCNA and YAP1 downstream genes CTGF and CYR61 mRNA expression in HepGLs organoids under different treatments. (L) Western blot and densitometric analysis of Ki67, PCNA, and nuclear YAP1 expression in HepGLs organoids under different treatments. The data represent the mean ± SEM. Statistical significance was assessed by two-tailed Student's t-test and two-way ANOVA. $*p < 0.05$, $**p < 0.01$, and $***p < 0.001$; n.s., not significant.

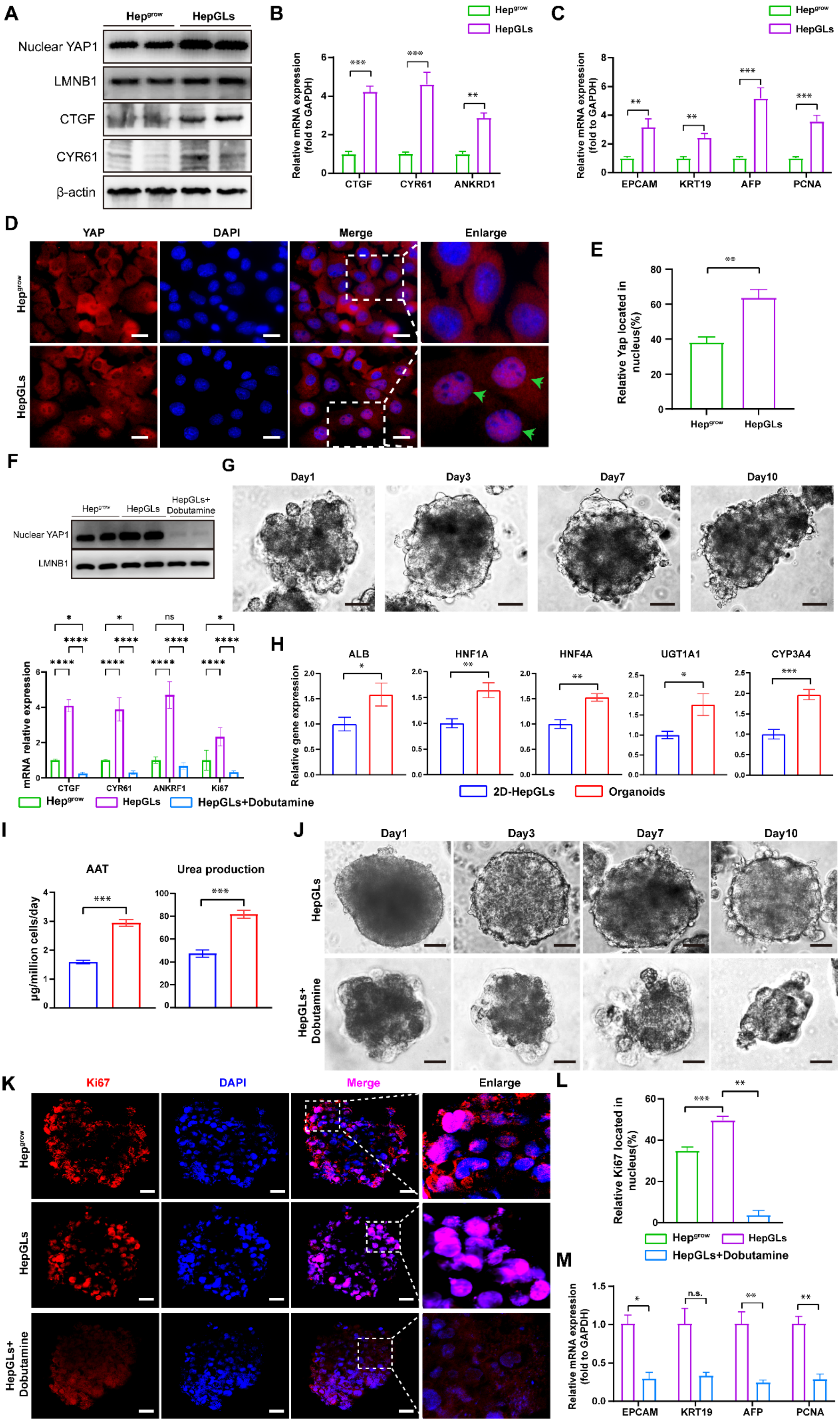
A
B
C
D
E
F
G
H
I
J
K
L
M
Nuclear YAP1
LMNB1
CTGF
CYR61
β-actin
HepGLs
YAP
DAPI
Merge
Enlarge
Day1
Day3
Day7
Day10
2D-HepGLs
Organoids
HepGLs+Dobutamine
ALB
HNF1A
HNF4A
UGT1A1
CYP3A4
AAT
Urea production
Ki67
EPCAM
KRT19
AFP
PCNA
ANKRD1
Relative mRNA expression (fold to GAPDH)
Relative Yap located in nucleus(%)
mRNA relative expression
Relative gene expression
μg/million cells/day
Relative Ki67 located in nucleus(%)

Figure S5. CCL2 promotes hepatocytes regeneration *in vitro* and *in vivo*

(A) Western blot analysis of nuclear YAP1, CTGF, and CYR61 protein levels in Hep^{grow} and HepGLs cells. (B) Real-time PCR analysis of mRNA expression levels of CTGF, CYR61, and ANKRD1 in Hep^{grow} and HepGLs cells (n=5 per group). (C) Real-time PCR analysis of mRNA expression levels of EPCAM, KRT19, and AFP in Hep^{grow} and HepGLs cells. (D-E) Immunofluorescence images and quantification of YAP localization in Hep^{grow} and HepGLs cells (scale bars, 100 µm). (F) Western blot analysis of nuclear YAP1 protein levels and CTGF, CYR61, ANKRD1 and ki67 mRNA levels in Hep^{grow} and HepGLs cells with or without Dobutamine (10 µmol/L). (G) Bright-field images of HepGLs cultured as organoids at different time points (scale bars, 100 µm). (H) Real-time PCR analysis of liver function-related gene expression in 2D-HepGLs and organoids (n=5 per group). (I) AAT secretion and urea production by 2D-HepGLs and organoids. (J) Bright-field images of HepGLs organoids cultured with or without Dobutamine treatment at different time points (scale bars, 100 µm). (K–L) Immunofluorescence images and quantification of Ki67 (red) expression in hepatic organoids (scale bars, 100 µm). (M) qPCR analysis of EPCAM, KRT19, AFP, and PCNA mRNA levels in HepGLs cells with or without Dobutamine treatment. Data are presented as mean ± SEM. Statistical significance was assessed using two-tailed Student's t-test and two-way ANOVA. *p < 0.05, **p < 0.01, and ***p < 0.001; n.s., not significant.

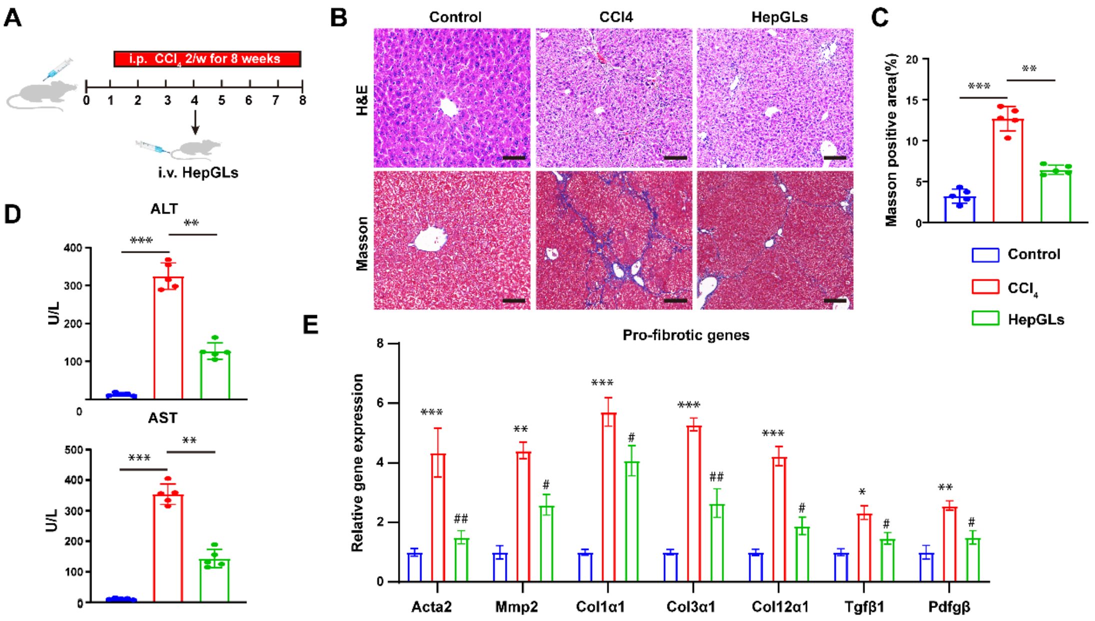

Figure S6. HepGLs effectively alleviate CCl_4-induced liver injury in NSG mice

(A) Schematic representation of the experimental design and timeline for CCl_4-induced liver injury in NSG mice treated with HepGLs for 8 weeks. (B) H&E and Masson staining of liver tissues from the indicated groups (scale bars, 100 µm; n=5 per group). (C) Quantification of Masson staining. (D) Serum ALT and AST levels in the indicated groups (n=5 per group). (E) Real-time PCR analysis of mRNA expression levels of pro-fibrotic genes in the three groups. The data represent the mean ± SEM. Statistical significance was assessed by two-tailed Student's t-test and two-way ANOVA. *$p < 0.05$, **$p < 0.01$, and ***$p < 0.001$; n.s., not significant.

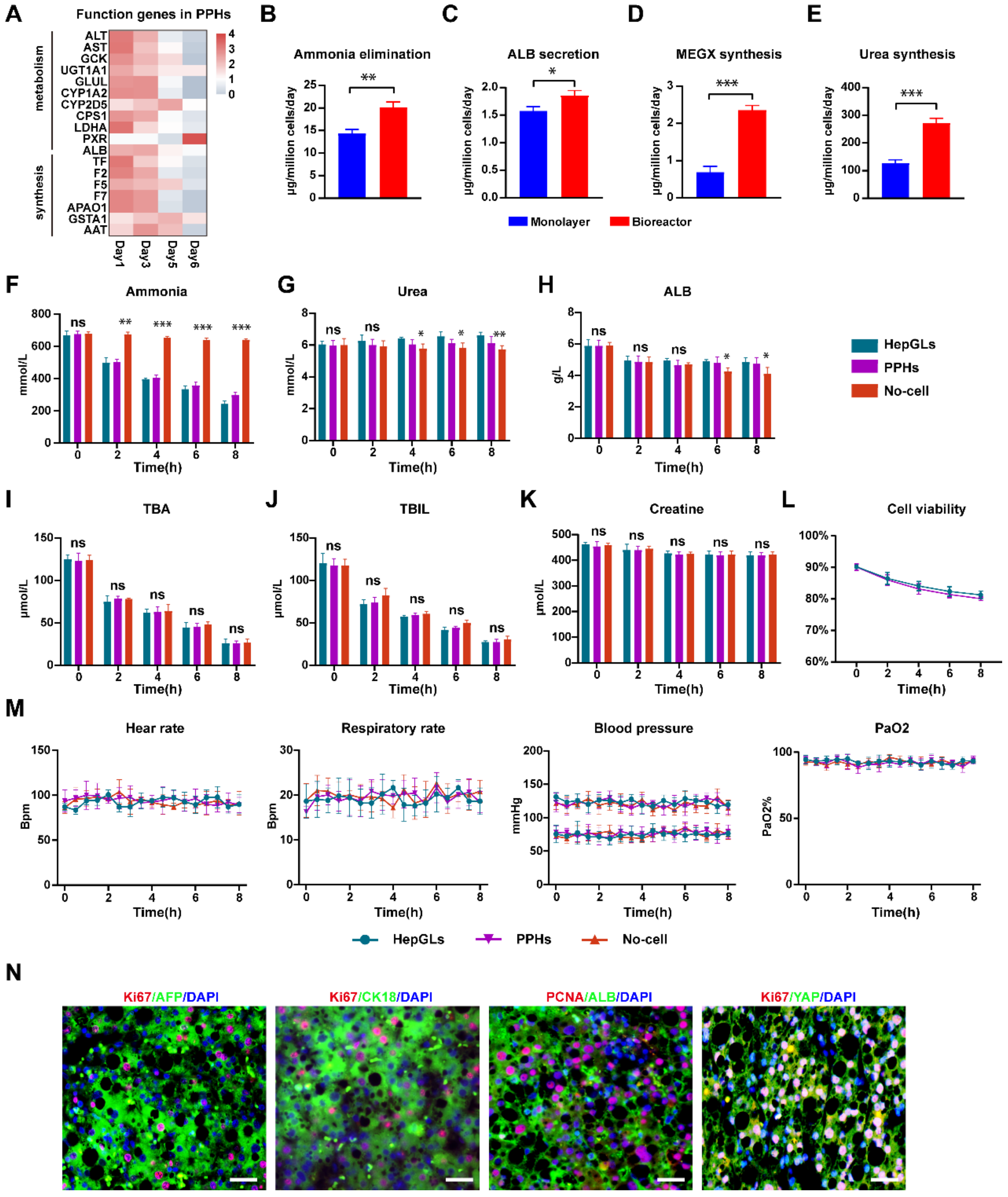

A
Function genes in PPHs
metabolism
synthesis
ALT
AST
GCK
UGT1A1
GLUL
CYP1A2
CYP2D5
CPS1
LDHA
PXR
ALB
TF
F2
F5
F7
APAO1
GSTA1
AAT
Day1
Day3
Day5
Day6
B
Ammonia elimination
C
ALB secretion
D
MEGX synthesis
E
Urea synthesis
μg/million cells/day
Monolayer
Bioreactor
F
Ammonia
G
Urea
H
ALB
mmol/L
g/L
Time(h)
HepGLs
PPHs
No-cell
I
TBA
J
TBIL
K
Creatine
L
Cell viability
μmol/L
M
Hear rate
Respiratory rate
Blood pressure
PaO2
Bpm
mmHg
PaO2%
N
Ki67/AFP/DAPI
Ki67/CK18/DAPI
PCNA/ALB/DAPI
Ki67/YAP/DAPI

Figure S7. BAL treatment of the preclinical porcine model of ALF

(A) PPHs' functional gene expression after 5 days of culture (n=3 per timepoint). (B-E) Ammonia elimination, ALB secretion, CYP450 metabolic activity assessed by monoethylglycinexylidide (MEGX) and Urea synthesis were evaluated in day-1 HepGLs cultured in the bioreactor in the indicated groups (n=3 per group). (F-K) Ammonia, urea, ALB, TBA, TBIL and Cr levels in simulated ALF serum (n=3). (L) PPHs and HepGLs viability in the BAL device over 8 h of circulation (n = 3 per time point). (M) Vital signs in the three groups during treatment (heart rate, respiratory rate, blood pressure and blood oxygen; n=5 per group). (N) Immunofluorescence images of Ki67, AFP, CK18, PCNA, ALB and YAP of the liver from the HepGLs group at 144 h (Scale bars, 100μm). The data represent the mean ± SEM. Statistical significance was assessed by two-tailed Student's t-test and two-way ANOVA. $*p < 0.05$, $**p < 0.01$, and $***p < 0.001$; n.s., not significant.

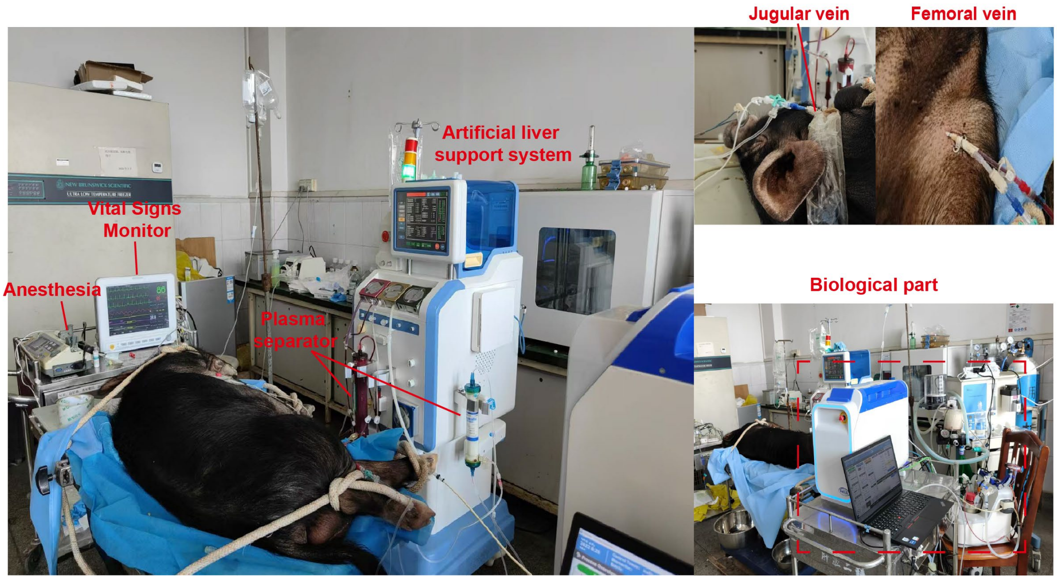

Figure S8. BAL treatment in an ALF porcine model

Supplementary Table

Supplementary Table.1 Antibodies.

Name	Supplier	Catalog No.
PCNA	Proteintech	10205-2-AP
AFP	Santa cruz	sc-130302
Albumin	Santa cruz	sc-374670
CYP3A4	Santa cruz	Sc-53850
Ki67	Invitrogen	PA5-114437
HNF4A	Abcam	ab92378
HNF1A	Nature Biosciences	A64618
SOX9	Nature Biosciences	A18886
AAT	ABclonal	A21972
TTR	ABclonal	A1120
ZO-1	Abcam	ab216880
Occludin	Abcam	ab216327
NGAL	Servicebio	GB111134
TLR4	Servicebio	GB15186
IBA	Nature Biosciences	A83118
GFAP	Servicebio	GB15096
TNFα	Servicebio	GB11188
CD206	UpingBioHANGZHOU	YP-Ab-14084
CCL2	Servicebio	GB11199
CK18	AntibodySystem SAS	PHC17801
YAP	Cell signaling technology	14074
CTGF	Nature Biosciences	A23678
CYR61	Nature Biosciences	A28875
Alexa Fluor® 488-conjugated AffiniPure Goat Anti-Mouse IgG H&L	MCE	HY-P8005
Alexa Fluor® 594-conjugated		

AffiniPure Goat Anti-Rabbit IgG H&L	MCE	HY-P8003
Alexa Fluor® 647-conjugated AffiniPure Goat Anti-Rabbit IgG H&L	MCE	HY-P80952

Supplementary Table.2 mRNA primers

Primer Name	Primer Sequence (5' to 3')
h-AFP-Forward Primer	CTTTGGGCTGCTCGCTATGA
h-AFP-Reverse Primer	GCATGTTGATTTAACAAGCTGCT
h-HNF1A-Forward Primer	CCATCCTCAAAGAGCTGGAG
h-HNF1A-Reverse Primer	GTGCTGCTGCAGGTAGGACT
h-ALB-Forward Primer	GAGACCAGAGGTTGATGTGATG
h-ALB-Reverse Primer	AGTTCCGGGGCATAAAAGTAAG
h-HNF1B-Forward Primer	GAGGAGGCATTCCGGCAAAA
h- HNF1B -Reverse Primer	TGTAAAACCGACTGGCTGGTC
h-HNF4A-Forward Primer	GATGTAGTCCTCCAAGCTCAC
h-HNF4A-Reverse Primer	GCCATCATCTTCTTTGACCCA
h-UGT1A1-Forward Primer	TTGTCTGGCTGTTCCCACTTA
h-UGT1A1-Reverse Primer	GGTCCGTCAGCATGACATCA
h-CYP3A4-Forward Primer	GTGGGGCTTTTATGATGGTCA
h-CYP3A4-Reverse Primer	ACATCTCCATACTGGGCAATGA
h-IL1b-Forward Primer	ATGATGGCTTATTACAGTGGCAA
h-IL1b-Reverse Primer	GTCGGAGATTCGTAGCTGGA
h-IL6-Forward Primer	ACTCACCTCTTCAGAACGAATTG
h-IL6-Reverse Primer	CCATCTTTGGAAGGTTCAGGTTG
h-TNFa-Forward Primer	CCTCTCTCTAATCAGCCCTCTG
h- TNFa -Reverse Primer	GAGGACCTGGGAGTAGATGAG
h-IFNG-Forward Primer	TCGGTAACTGACTTGAATGTCCA
h-IFNG-Reverse Primer	TCGCTTCCCTGTTTTAGCTGC
h-CCL2-Forward Primer	CAGCCAGATGCAATCAATGCC
h-CCL2-Reverse Primer	TGGAATCCTGAACCCACTTCT
h-CCL4-Forward Primer	CTGTGCTGATCCCAGTGAATC
h-CCL4-Reverse Primer	TCAGTTCAGTTCCAGGTCATACA
h-IL4-Forward Primer	CCAACTGCTTCCCCCTCTG
h-IL4-Reverse Primer	TCTGTTACGGTCAACTCGGTG

h-TGFB-Forward Primer	GGCCAGATCCTGTCCAAGC
h-TGFB-Reverse Primer	GTGGGTTTCCACCATTAGCAC
h-GAPDH-Forward Primer	TCGGAGTGAACGGATTTGGC
h-GAPDH-Reverse Primer	TGACAAGCTTCCCGTTCTCC
